# Combined genome and transcriptome sequencing to investigate the plant cell wall degrading enzyme system in the thermophilic fungus *Malbranchea cinnamomea*

**DOI:** 10.1186/s13068-017-0956-0

**Published:** 2017-11-13

**Authors:** Silvia Hüttner, Thanh Thuy Nguyen, Zoraide Granchi, Thomas Chin-A-Woeng, Dag Ahrén, Johan Larsbrink, Vu Nguyen Thanh, Lisbeth Olsson

**Affiliations:** 1 Department of Biology and Biological Engineering, Division of Industrial Biotechnology, Chalmers University of Technology, 412 96 Gothenburg, Sweden; 2 Wallenberg Wood Science Center, Chalmers University of Technology, 412 96 Gothenburg, Sweden; 3 Centre for Industrial Microbiology, Food Industries Research Institute, Thanh Xuan, Ha Noi, Vietnam; 4 GenomeScan B.V., Plesmanlaan 1/D, 2333 BZ Leiden, The Netherlands; 5 National Bioinformatics Infrastructure Sweden (NBIS), Institute of Biology, Lund University, 223 62 Lund, Sweden

**Keywords:** Plant biomass, Carbohydrate-active enzymes, Cellulase, Wheat bran, Xylan, *Malbranchea pulchella*

## Abstract

**Background:**

Genome and transcriptome sequencing has greatly facilitated the understanding of biomass-degrading mechanisms in a number of fungal species. The information obtained enables the investigation and discovery of genes encoding proteins involved in plant cell wall degradation, which are crucial for saccharification of lignocellulosic biomass in second-generation biorefinery applications. The thermophilic fungus *Malbranchea cinnamomea* is an efficient producer of many industrially relevant enzymes and a detailed analysis of its genomic content will considerably enhance our understanding of its lignocellulolytic system and promote the discovery of novel proteins.

**Results:**

The 25-million-base-pair genome of *M. cinnamomea* FCH 10.5 was sequenced with 225× coverage. A total of 9437 protein-coding genes were predicted and annotated, among which 301 carbohydrate-active enzyme (CAZyme) domains were found. The putative CAZymes of *M. cinnamomea* cover cellulases, hemicellulases, chitinases and pectinases, equipping the fungus with the ability to grow on a wide variety of biomass types. Upregulation of 438 and 150 genes during growth on wheat bran and xylan, respectively, in comparison to growth on glucose was revealed. Among the most highly upregulated CAZymes on xylan were glycoside hydrolase family GH10 and GH11 xylanases, as well as a putative glucuronoyl esterase and a putative lytic polysaccharide monooxygenase (LPMO). AA9-domain-containing proteins were also found to be upregulated on wheat bran, as well as a putative cutinase and a protein harbouring a CBM9 domain. Several genes encoding secreted proteins of unknown function were also more abundant on wheat bran and xylan than on glucose.

**Conclusions:**

The comprehensive combined genome and transcriptome analysis of *M. cinnamomea* provides a detailed insight into its response to growth on different types of biomass. In addition, the study facilitates the further exploration and exploitation of the repertoire of industrially relevant lignocellulolytic enzymes of this fungus.

**Electronic supplementary material:**

The online version of this article (10.1186/s13068-017-0956-0) contains supplementary material, which is available to authorised users.

## Background

Conventional resources, such as oil, gas and coal, used in the production of fuels and chemicals are becoming limited and are the cause of many environmental problems. The alternative use of renewable and abundant (ligno)cellulosic materials, i.e. plant cell walls, as feedstocks in second-generation biorefineries has consequently been the subject of intense research and innovation during recent decades [1–3]. Microbial cells and their enzymes are here utilised to convert low-value biomass into valuable products such as biofuels and biochemicals. Still, efficient saccharification of the lignocellulosic material remains challenging. Lignocellulose consists of a heterogeneous matrix comprising crystalline cellulose microfibrils enmeshed in a matrix of various types of hemicelluloses and pectins, and in addition contains significant amounts of the polyaromatic structure lignin [4]. A large number of enzymatic activities are required to fully deconstruct plant biomass, most of which are catalogued in the carbohydrate-active enzymes database (CAZy) [5–7]. These carbohydrate-active enzymes (CAZymes) are involved in the assembly and breakdown of complex carbohydrates and glycoconjugates, and have been classified into glycoside hydrolases (GHs), glycosyltransferases (GTs), polysaccharide lyases (PLs), carbohydrate esterases (CEs), enzymes with auxiliary activities (AAs) and carbohydrate-binding modules (CBMs).

Fungi are main degraders of plant biomass in nature and fungal CAZymes from both Ascomycetes and Basidiomycetes are widely used in industrial processes [8–10]. Most enzymes used today in biomass conversion are mesophilic, but higher process temperatures could enable faster reaction rates, lower viscosity, better cell wall disintegration and enzyme penetration into the raw material, increased mass transfer and reduced risk of contamination [11–14]. Therefore, thermophilic organisms, with growth optima between 45 and 80 °C [15], are promising sources of thermostable enzymes and have hitherto not been as extensively explored as their mesophilic counterparts [16].

The thermophilic fungus *Malbranchea cinnamomea* belongs to the order of Onygenales [17], can grow at temperatures over 50 °C and is able to utilise many different types of plant biomass, including rice straw, sorghum, corn cob, wheat bran, coconut meal and *Parthenium hysterophorus* (carrot grass), as well as crystalline cellulose [16, 18–25]. When grown on carrot grass and rice straw, *M. cinnamomea* has been found to be the most efficient source of GHs among nine thermophilic fungi tested [26]. Characterised enzymes from *M. cinnamomea* include a β-mannanase [18], an alkaline β-1,3-1,4-glucanase (lichenase) [19], an α-amylase [20], an α-glucosidase [21], xylanases [22–24] and a cutinase [25], all of which have been reported to have temperature optima between 45 and 80 °C (Additional file 1). A proteomics analysis of *M. cinnamomea* CM-10T, using the sequence of *M. cinnamomea* strain CBS 343.55 [27], revealed a large array of enzymes from major GH families involved in efficient biomass degradation [26], but to the best of our knowledge no detailed genome analyses have been published to date. We here expand the existing knowledge of *M. cinnamomea* with a combined genome sequencing and transcriptomic analysis of strain FCH 10.5 to provide a comprehensive view of its lignocellulolytic capabilities. We identified genes differentially expressed during growth on beechwood xylan and wheat bran, which indicate differences in the strategy of the fungus to deconstruct hardwood and cereal hemicelluloses. The data presented here will form a basis for systematic exploration of the full potential of *M. cinnamomea* as a source of thermostable enzymes.

## Methods

### Fungal isolation, identification and cultivation

The fungus used in the present study was isolated from compost at the Cau Dien municipal waste treatment factory in Hanoi, Vietnam [geographical location 21°01′01.7″N, 105°45′09.2″E, 11 m (longitude, latitude, altitude)], and was grown on potato dextrose agar (PDA) at 50 °C. For DNA extraction and species identification, one loopful of mycelium was transferred to a microcentrifuge tube containing 1 mL 2X SSC (15 mM sodium citrate, 150 mM NaCl, pH 7.0) and heated to 99 °C for 10 min. Cells were collected by centrifugation (10,000*g*, 1 min), the supernatant was discarded, and 100 μL of glass beads (0.2–0.5 mm in diameter; Roth, Germany), 100 μL phenol–chloroform (25:24:1, saturated with 10 mM TrisHCl, pH 8.0) and 150 μL water were added to the cell pellet. The cells were disrupted using a Mini-Beadbeater-8 (Biospec, USA) for 45 s, then centrifuged (14,000*g*, 10 min), and the upper layer of liquid was transferred to a new tube. The DNA solutions were purified using a Silica Bead DNA Gel Extraction kit (Thermo Scientific), according to the manufacturer’s instructions. The resulting DNA was used to amplify the internal transcribed spacer (ITS) regions using the universal primers ITS1 (forward; 5′ TCCGTAGGTGAACCTGCGG 3′) and ITS4 (reverse; 5′ TCCTCCGCTTATTGATATGC 3′) [28]. Amplicons were sequenced, compared to available databases using BLAST and the fungus was identified as *M. cinnamomea*. The ITS sequence of FCH 10.5 was compared with ITS sequences of other *M. cinnamomea* strains in Genbank by multiple sequence alignment (see Additional file 2). The strain FCH 10.5 has been deposited at the CBS-KNAW fungal culture collection [29] and the ITS sequence has been deposited at NCBI Genbank under the Accession Number MF838862.1.

### Growth on plates with different carbon sources

*M. cinnamomea*, *Myceliophthora thermophila*, *Aspergillus oryzae*, *Phanerochaete chrysosporium* and *Schizophyllum commune* (all from our own culture collection, except *A. oryzae* JCM 2239 from the Japan Collection of Microorganisms) were grown on plates containing a basal medium (4 g L^−1^ KH_2_PO_4_, 13.6 g L^−1^ (NH_4_)_2_SO_4_, 0.8 g L^−1^CaCl_2_·2H_2_O, 0.6 g L^−1^ MgSO_4_·7H_2_O, 6 g L^−1^ Bacto peptone, 10 mg L^−1^ FeSO_4_·7H_2_O, 3.2 mg L^−1^ MnSO_4_·H_2_O, 2.8 mg L^−1^ ZnSO_4_·7H_2_O, 4 mg L^−1^ CoCl_2_·6H_2_O, 200 mL L^−1^ Tween 80), and 15 g L^−1^ agar. The pH was adjusted to 5.8 and carbon sources were added before autoclaving. As carbon source, 1% of Avicel, beechwood xylan, starch, guar gum, gum arabic, carboxymethyl cellulose (CMC), citrus pectin, chitosan, locust bean gum or inulin from Dahlia tubers, or 0.5% of cellobiose, d-galactose, d-glucose, d-mannose or d-xylose, respectively, was added. All chemicals were obtained from Sigma-Aldrich, except d-glucose from Merck and d-xylose from Fluka. Plates were inoculated with spores and grown at 30 °C or 50 °C for 7 days.

### Enzyme activity assays

Liquid cultures of *M. cinnamomea* FCH 10.5 were grown in 125 mL liquid medium in baffled Erlenmeyer flasks (500 mL), containing the basal medium supplemented with 1% wheat bran or 1% beechwood xylan. The liquid cultures were inoculated with a spore suspension prepared from a fresh PDA plate. Cultures were grown at 50 °C for 9 days with shaking at 250 rpm, and samples taken every 24 h. For determination of xylanase or endoglucanase activity, 0.1 mL of crude culture filtrate was mixed with 0.2 mL of substrate (1% xylan or 1% CMC). After incubation at 50 °C for 20 min, 0.6 mL DNS reagent, consisting of 1% (w/v) dinitrosalicylic acid (DNS), 0.2% (w/v) phenol and 1% (w/v) NaOH, were added and kept at 95 °C for 5 min. The sample was cooled down, 0.4 mL were mixed with 1.8 mL water and the absorbance was measured at 540 nm. Standard curves were prepared with xylose and glucose.

### DNA and RNA extraction

Cultivation of fungal mycelium for genome sequencing was carried out in 125 mL liquid medium in baffled Erlenmeyer flasks (500 mL) at 50 °C for 48 h on a shaking incubator at 250 rpm in basal medium with glucose as the carbon source (20 g L^−1^). The liquid culture was inoculated with a spore suspension prepared from a fresh PDA plate. After 2 days of cultivation, mycelium was harvested by filtering through Miracloth (Merck Millipore) in a Büchner filter. After pressing between paper towels, the mycelium was snap frozen in liquid nitrogen and stored at − 80 °C until DNA extraction. Cells were broken using a tissue lyser with tungsten steel balls, pre-cooled in liquid nitrogen, for 30 s at full speed. Immediately, 20 mL CTAB buffer (2% CTAB, 100 mM TrisHCl, pH 8.0, 20 mM EDTA, 1.4 M NaCl) were added to ~ 5 mL mycelium powder. After incubation at 57 °C for 1 h, cell debris was spun down (10,000 *g*, 10 min, 4 °C) and DNA was purified from the supernatant by phenol–chloroform extraction and isopropanol precipitation [30]. The final DNA pellet was resuspended in 1 mL TE buffer (10 mM TrisHCl, pH 8.0, 1 mM EDTA). After incubation at 60 °C for 2 h with RNAse A (200 mg/mL), DNA was again extracted by phenol–chloroform extraction, precipitated with isopropanol and purified in a further step with the DNeasy Plant Mini Kit (Qiagen), according to the manufacturer’s instructions. Quality of the purified DNA was verified by agarose gel electrophoresis, Nanodrop (Thermo Scientific) and Qubit (Life Technologies) before genome sequencing.

For RNA extraction, 2 × 200 mL of pre-culture were cultivated for 2 days (in 1000-mL baffled Erlenmeyer flasks) in glucose-containing liquid medium, as described above, after which the mycelium was filtered and extensively washed with medium lacking a carbon source. A total of 15 g of mycelium was then divided equally between six 250-mL baffled Erlenmeyer flasks containing 50 mL basal liquid medium supplemented with either 10 g L^−1^ glucose, wheat bran or beechwood xylan as the sole carbon sources. Two independent duplicate experiments were performed for each substrate. After 4 and 48 h of incubation at 50 °C on a shaking incubator, total RNA was extracted using TRIzol (Invitrogen) and chloroform, and further purified with the RNeasy Plant RNA Kit with on-column DNAse digestion. The quality of the purified RNA was verified by agarose gel electrophoresis, Nanodrop (Thermo Scientific) and Qubit (Life Technologies). Subsequent RNA sequencing was performed with a 1:1 mixture of RNA samples isolated at 4 and 48 h, to increase the chances of detecting both early- and late-response genes in the differential expression analysis. However, the transcriptional response of genes induced only at the early or later time point may be diluted.

### Genome sequencing and assembly

The NEBNext Ultra DNA Library Prep kit for Illumina (New England Biolabs) was used to process the samples according to the manufacturer’s protocol. Fragmentation of DNA was carried out using a Covaris ultrasonicator (Thermo Scientific). The quality and yield after sample preparation were measured using Bioanalyzer (Agilent Technologies). Clustering and DNA sequencing were performed using the Illumina cBot and HiSeq 2500 systems with a DNA concentration of 8.0 pM, using 250-bp paired-end reads. Procedures were carried out by GenomeScan B.V., Leiden. Image analysis, base calling and quality check were performed with the Illumina data analysis pipelines RTA v1.18.64 and Bcl2fastq v1.8.4. For adapter trimming, presumed adapter sequences were removed from the read when the bases matched a sequence in the TruSeq adapter sequence set with 2 or fewer mismatches and an alignment score of at least 12. Raw data were filtered and clipped based on base quality scores; bases with Phred quality scores below Q22 were removed and reads containing these bases were split and removed when shorter than 36 bp. A short-read genome assembler based on De Bruijn graphs, Abyss v1.3.7 [31, 32], with a k-mer length of 64 was used for assembly. Scaffolds shorter than 500 bp were removed. The *M. cinnamomea* FCH 10.5 whole genome assembly has been submitted to the European Nucleotide Archive and deposited at DDBJ/EMBL/GenBank under the assembly Accession Number FQSS02000000.

### Transcriptome analysis

The NEBNext Ultra Directional RNA Library Prep Kit for Illumina (New England Biolabs) was used to process the samples according to the manufacturer’s instructions. Briefly, mRNA was isolated from total RNA using oligo-dT magnetic beads and used to synthesise cDNA. The cDNA was ligated with sequencing adapters and PCR amplified. The quality and yield after sample preparation were determined with the Fragment Analyzer (Advanced Analytical). The size of the resulting products was consistent with the expected size distribution (a broad peak between 300 and 500 bp). Standard Illumina primers for Illumina cBot and HiSeq 2500, and the HiSeq control software HCS v2.2.58 were used according to the manufacturer’s protocols for clustering and DNA sequencing with a concentration of 16.0 pM. The Illumina data analysis pipelines RTA v1.18.64 and Bcl2fastq v2.17 were used for image analysis, base calling and quality check. Sequencing was performed on an Illumina HiSeq 2500 sequencer. The assembled genome from the DNA sequencing was used as a reference to map the reads using the packages Tophat (v2.0.14. Linux_x86_64) and Bowtie (v2-2.1.0) with a default mismatch rate of 2%. The frequency with which a read was mapped on a transcript was determined based on the mapped locations from the alignment. To normalise for transcript length, fpkm (fragments per kilobase of transcript per million mapped reads) were calculated. For differential expression analysis, the read counts were loaded into the DESeq package v 1.10.1 [31]. Genes were considered differentially expressed if they showed a log2 fold change ≥ 1 and the adjusted *p* value was < 0.05. The reproducibility of biological replicates is shown in Additional file 2.

### Gene prediction and annotation

Gene prediction was performed ab initio using the HMM-based algorithm Glimmer v. 3.02 [33], which was trained using the genome of *Uncinocarpus reesii* (downloaded from JGI, [34]). Additionally, the software tool CodingQuarry [35] was used for an evidence-based method of gene finding, where exon–intron boundaries were determined using the mapped mRNA-seq reads. For functional gene annotation, coding DNA sequences (CDS) were translated into amino acid sequences and a BLASTp search (version 2.2.28+) [36] was performed on the UniprotKB/Swiss-prot database with default parameters (*E* value cut-off of 1, similarity cut-off of 30%). Classification into gene ontology (GO) categories and InterProScan were performed with Blast2GO software [37]. GO enrichment tests were performed with the R package Piano [38]. Secretory proteins were predicted using the SignalP 4.1 Server [39] or the WoLF PSORT algorithm [40]. Genes containing CAZy domains were identified using dbCAN release 5.0 [41] which searches CAZy family-specific HMMs with HMMER3, and NCBI’s conserved domain database CDD [42].

## Results

### Strain identification and growth on different carbohydrates

**Fig. 1:**
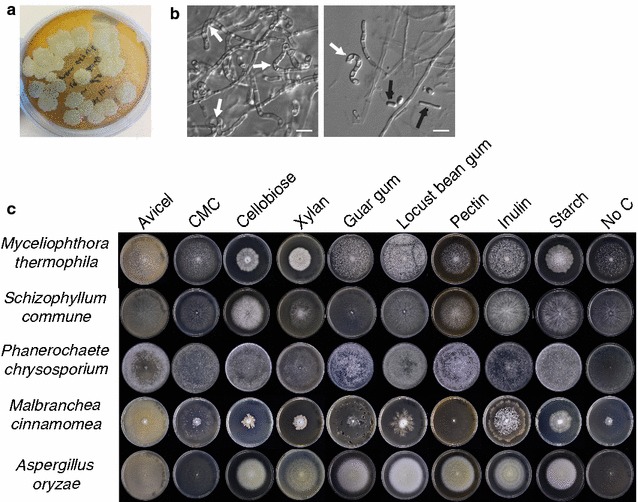
Growth of *M. cinnamomea* on different carbon sources. **a** *M. cinnamomea* grown for 4 days on PDA agar. **b** Microphotographs of *M. cinnamomea* FCH 10.5 growing at 50 °C on basal media agar with glucose as the carbon source. Curved fertile hyphae (white arrows) and arthroconidia (black arrows) are visible. **c** Growth of *M. cinnamomea* FCH 10.5, and four other filamentous fungi, on cellulose [Avicel, carboxymethyl cellulose (CMC)], cellobiose, plant polysaccharides (beechwood xylan, guar gum, locust bean gum, citrus pectin, inulin, starch) and no carbon (no C). All fungi were grown for 7 days, either at 30 °C (*S. commune*, *P. chrysosporium*, *A. oryzae*) or 50 °C (*M. thermophila*, *M. cinnamomea*)

Strain FCH 10.5, isolated from compost at a municipal waste treatment plant in Hanoi and identified as *M. cinnamomea* by ITS sequencing, grew well on PDA at 50 °C and formed yellow, powdery colonies of irregular shape and produced curved, fertile hyphae and arthroconidia (Fig. 1a, b), as described previously [43]. A comparison of ITS sequences to other *M. cinnamomea* strains in the NCBI Genbank database revealed a very high sequence identity of strain FCH 10.5 to CM-10T, isolated from composting soil near Punjab, India, which had been the subject of an earlier proteomics analysis [26] (Additional file 3).

Previous studies have shown that *M. cinnamomea* has high cellulolytic and hemicellulolytic activities when cultivated on wheat bran, rice straw, sorghum straw or cellulose [16, 27, 44]. We also detected xylanase and endoglucanase activities in liquid cultures of FCH 10.5 grown on wheat bran or beechwood xylan (Additional file 4). During growth on xylan, higher xylanase activities were observed than during growth on wheat bran, while cultures on wheat bran showed a higher endoglucanase activity. The peak of enzymatic activity was reached at between 6 and 8 days of cultivation.

The growth of *M. cinnamomea* FCH 10.5 on various, mono-, di-, and polysaccharides was evaluated in comparison to a selection of both thermophilic and mesophilic strains (Fig. 1c and Additional file 5). The preferred substrates of *M. cinnamomea* FCH 10.5 among the tested ones were beechwood xylan, cellobiose and starch. Good growth could also be observed on inulin and locust bean gum (galactomannan with galactose residues on every fourth mannose), while only poor growth was observed on guar gum (galactomannan with galactose residues on every second mannose) and cellulose (Avicel, CMC). No growth was detected on citrus pectin as the sole carbon source.

### Genome characteristics

The whole genome sequence of *M. cinnamomea* FCH 10.5 was determined by sequencing on the Illumina HiSeq 2500 platform. Libraries of 300- to 500- and 500- to 800-bp fragments were sequenced to produce 250-bp paired-end reads. This produced raw yields of 3.5 and 2.6 Gb, respectively. The quality was determined as the percentage of bases with a *Q* score ≥ 30, and was greater than 88% in both cases. The final genome size of the assembly was 24.96 Mb comprising 797 scaffolds. The output of two gene prediction algorithms (Glimmer and CodingQuarry) was combined into a single gene model and yielded 9437 protein-coding genes with an average length of 388 amino acids. Both the size of the assembled *M. cinnamomea* sequence and the number of predicted protein-coding genes fell into the range of other sequenced fungal genomes [45]. Species in the order Onygenales have genome sizes of about 25 Mb and contain on average around 8700 protein-coding genes [46]. The GC content of *the M. cinnamomea* genome was determined to be 53.4% for coding regions and 49.8% for the overall genome. To better describe integrity of the genome assembly, Benchmarking Universal Single-Copy Orthologs (BUSCO) analysis was performed, a method for quantitative assessment of genome assembly and annotation completeness [47]. The presence of the BUSCO set of 1438 well-conserved, fungal single-copy genes was determined in the genome of FCH 10.5. Of those, 1357 were found to be complete (C: 94%), indicating an assembly of excellent integrity. Additional file 6 summarises the *M. cinnamomea* FCH 10.5 genome properties.

**Fig. 2:**
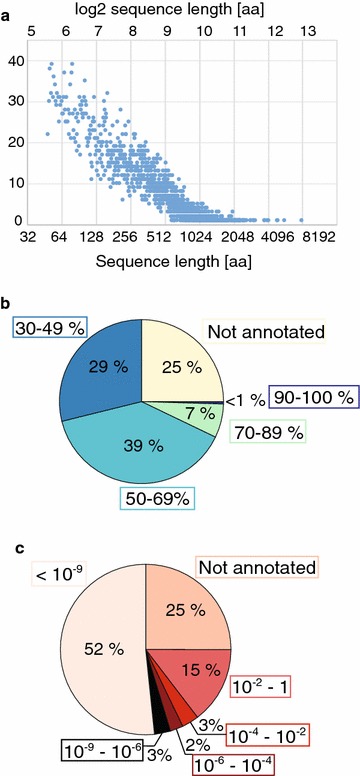
Statistics from the annotation of predicted proteins using BLASTp. **a** Length distribution of the 9437 predicted proteins. Similarity (in %) **b** and *E* values **c** of BLASTp hits to annotated proteins in the UniprotKB/Swiss-prot database

Average, shortest and longest sizes of the 9437 predicted proteins were 338, 49 and 8085 amino acids, respectively (Fig. 2a), with the majority of proteins being between 143 (25th percentile) and 514 (75th percentile) amino acids long. With the selected parameters, 25% of the predicted proteins could not be annotated by BLASTp. Among the proteins with BLASTp hits, 0.45% showed a similarity of over 90% to proteins in the UniprotKB/Swiss-prot database, 6.65% a similarity of between 70 and 89, and 39.01% a similarity of 50–69% (Fig. 2b, Additional file 6). Over half of the protein sequences of *M. cinnamomea* could be annotated with an *E* value of below 10^−9^ (Fig. 2c), which indicates high confidence in the annotation results. A GO classification was performed with Blast2GO which divided the annotated proteins into 36 functional groups representing the categories biological process, cellular component and molecular function (Additional file 7).

### Carbohydrate-active enzymes in *M. cinnamomea*

A total of 301 CAZy domains were found, among which were 137 GHs, 62 GTs, 4 PLs, 24 CEs, 42 AAs and 32 CBMs from a total of 108 different families (Table 1, Additional file 8). A number of fungal species were chosen as a comparison to the CAZyme repertoire of *M. cinnamomea* (a phylogenetic tree showing their evolutionary relation can be found in Additional file 9), including well-known plant cell wall degraders from Ascomycetes and Basidiomycetes. The importance of complex carbohydrates as nutrients for *M. cinnamomea* is apparent when its CAZyme content is compared to the yeasts *Kluyveromyces lactis*, *Saccharomyces cerevisiae* and *Yarrowia lipolytica,* which have only about a third of the number of total CAZymes of *M. cinnamomea* and which primarily consume monosaccharides. Among fungi that utilise plant cell walls, the Ascomycetes *Neurospora crassa and Myceliophthora* *thermophila*, as well as the Basidiomycete *Phanerochaete chrysosporium* and the Zygomycete *Rhizopus* *oryzae* contain very similar numbers of CAZymes in their genomes to *M. cinnamomea* (Table 1). The distribution of CAZyme members across the GH families was quite different, however, with for instance *R. oryzae* [48], a known pectin degrader, possessing 18 GH28 family members, while *M. cinnamomea* has none (Additional file 8). *Phanerochaete chrysosporium* has five GH28 members, and *M. thermophila* has two, and both showed growth on pectin, in contrast to *M. cinnamomea* (Fig. 1c). *Myceliophthora thermophila* (thermophile) and *N.* *crassa* (mesophile) have similar distributions of CAZy families to *M. cinnamomea* in most categories, with the exception of the CBM1 family, where *M. cinnamomea* has only one member, and *N. crassa* and *M. thermophila* have 19 and 16, respectively (Additional file 8). Fungal species phylogenetically closer to *M. cinnamomea*, such as *Aspergillus* *niger*, *Aspergillus* *nidulans*, *Aspergillus.* *oryzae* and *Penicillium* *chrysogenum*, also share a similar CAZy family distribution with *M.* *cinnamomea*, but possess roughly twice the number of members in many GH families, such as GH2, 5, 11, 12, 13 and 43. However, *M.* *cinnamomea* possesses a comparatively high number of AAs and is especially enriched in families AA7, 9 and 11.

**Table 1:** Comparison of the numbers of CAZymes in *M. cinnamomea* with those in other fungi

Phylum	Species	GH	GT	PL	CE	AA	CBM	All
ASC	*Yarrowia lipolytica* ^a^	42	45	0	1	10	8	106
	*Saccharomyces cerevisiae* ^a^	45	65	0	2	6	15	133
	*Kluyveromyces lactis* ^a^	47	63	0	1	0	11	122
	*Arthrobotrys oligospora* ^a^	205	87	15	30	33	180	550
	*Malbranchea cinnamomea* ^b^	137	62	4	24	42	32	301
	*Penicillium chrysogenum* ^a^	222	103	9	22	22	51	429
	*Aspergillus nidulans* ^a^	264	92	21	31	33	44	485
	*Aspergillus niger* ^a^	253	121	8	23	68	55	528
	*Aspergillus oryzae* ^a^	307	117	23	29	30	37	543
	*Leptosphaeria maculans* ^a^	220	98	19	33	33	53	456
	*Thielavia terrestris* ^a^	212	91	4	28	58	80	473
	*Myceliophthora thermophila* ^a^	195	87	8	28	50	50	418
	*Podospora anserina* ^a^	213	92	8	41	105	104	563
	*Neurospora crassa* ^a^	174	78	4	22	35	42	355
	*Magnaporthe grisea* ^a^	265	105	4	52	92	114	632
	*Fusarium graminearum* ^a^	253	110	21	45	72	87	588
BAS	*Rhodosporidium toruloides* ^a^	75	101	4	10	17	11	218
	*Sporisorium reilianum* ^a^	104	66	3	12	21	6	212
	*Schizophyllum commune* ^c,d^	237	77	5	40	70	39	468
	*Piriformospora indica* ^a^	206	73	16	45	57	141	538
	*Phanerochaete chrysosporium* ^e,f^	170	70	1	20	81	71	413
	*Postia placenta* ^g,h^	174	102	2	22	22	22	344
ZYG	*Rhizopus oryzae* ^i,j^	123	145	6	48	10	38	370
OOM	*Phytophthora infestans* ^*a*^	283	157	67	21	0	37	565

*ASC* Ascomycota, *BAS* Basidiomycota, *ZYG* Zygomycota, *OOM* Oomycota

^a^[7]; ^b^this study; ^c^[76]; ^d^[77]; ^e^[78]; ^f^[79]; ^g^[80]; ^h^[81]; ^i^[48]; ^j^[82]

The concerted action of members from various CAZy families is required for the efficient degradation of plant cell wall polymers. For example, β-1,4-endoglucanases, cellobiohydrolases, β-glucosidases and lytic polysaccharide monooxygenases (LPMOs) are needed for the deconstruction of cellulose [49]. These types of enzymes have fungal representatives in the nine CAZy families GH1, 3, 5, 6, 7, 12 and 45, and AA9 and 10. Putative candidates for all four cellulase types spanning eight different CAZy families were found in the *M. cinnamomea* FCH 10.5 genome, indicating that the fungus is theoretically capable of converting cellulose into monosaccharides (Additional file 8), contrasting the poor growth observed on plates containing Avicel or CMC as the only carbon source (Fig. 1c). Closer inspection of the CAZy families involved in the hydrolysis of other plant cell wall polymers revealed that *M. cinnamomea* appears particularly well equipped for the degradation of xylan and chitin. In addition, the fungus probably possesses the genetic repertoire necessary for the deconstruction of xyloglucan, glucomannan and starch (Additional file 8). Surprisingly, no predicted α-1,6-galactosidase was found in the genome, even though *M. cinnamomea* grew well on locust bean galactomannan (Fig. 1c), suggesting that other enzymes might be involved in hydrolysis of this polymer. Regarding pectin, no proteins harbouring a GH28 domain were found to be present in the genome. The CAZy family GH28 contains pectin-degrading activities such as rhamnogalacturonan hydrolase, polygalacturonase, galacturan 1,4-α-galacturonidase, exo-poly-α-galacturonosidase and rhamnogalacturonan hydrolase [50]. However, pectate/pectin lyases (family PL1), a rhamnogalacturonan lyase (family PL4), a pectate disaccharide lyase (family PL3) and a rhamnoglacturonyl hydrolase (family GH105) were found, as well as several pectin esterases (families CE8 and 12) (Additional file 8).

### Multimodular CAZymes in the genome of *M. cinnamomea*

**Fig. 3:**
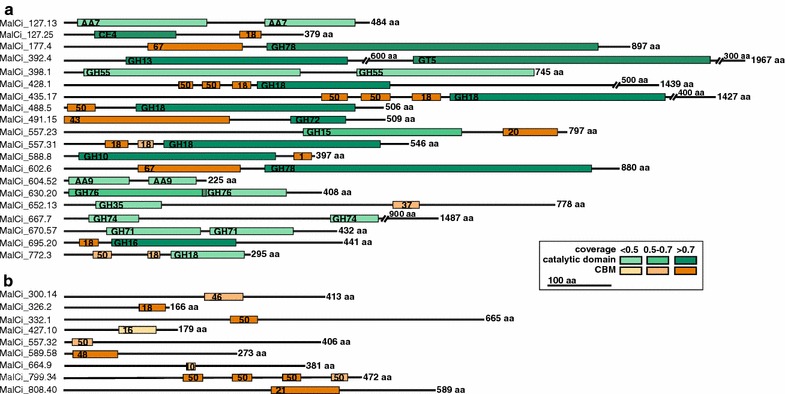
Proteins with more than one CAZy domain or with only a CBM domain, but no predicted catalytic domain. Gene ID is shown to the left, and the total length of the predicted protein to the right. The coloured bars indicate the location of the CAZy domains along the protein sequence; catalytic domains are green and CBMs are orange. The numbers in the bars indicate the CAZy family as classified in the CAZy database. The intensity of the shading indicates the coverage between query and subject in the dbCAN search. **a** Twenty putative proteins with at least one catalytic CAZy domain in addition to one or several CBM domains were found in the *M. cinnamomea* genome. **b** Eight putative proteins without predicted catalytic domains, but one or more CBM domains were present in the genome

CAZymes frequently exist as modular enzymes containing multiple CAZy domains, most often as a combination of a catalytic domain and a CBM. On average, 40% of fungal cellulases and hemicellulases are modular enzymes where the catalytic domain is connected to a non-catalytic CBM, to prolong contact with target polysaccharides [51, 52]. Twenty-one genes were found in *M.* *cinnamomea* that contained two or more predicted CAZy domains (Fig. 3). Of these, 13 genes had one catalytic domain in addition to one, two or three CBMs. Seven genes had a combination of two or three catalytic domains and one gene was found to contain four CBM50 domains. Although a CBM1 domain is fairly abundant in other fungal species, in particular in cellulase enzymes [52], in *M. cinnamomea* FCH 10.5 this domain could only be detected in one gene, fused to a GH10 domain. In addition to multimodular CAZymes, eight genes were found for which searches in the dbCAN and the NCBI CDD database resulted in hits for CBMs, but not for conserved catalytic domains (Fig. 3).

### CAZymes expressed during growth on glucose, wheat bran and xylan

To investigate how gene expression is influenced by growth on an easily metabolised carbon source, where mostly constitutive genes are predicted to be expressed compared to growth on a more complex carbon sources, RNAseq analysis was conducted on *M. cinnamomea* cultivated on glucose, wheat bran and beechwood xylan. Beechwood xylan, comprising a xylan backbone with 4-*O*-methyl glucuronic acid side groups, was chosen to study the expression of genes specific for hardwood hemicellulose degradation, while wheat bran was chosen as a cereal substrate of higher complexity, predicted to induce the expression of genes encoding a variety of CAZymes. A total of 266 distinct transcripts containing CAZy domains (of the 271 CAZy domain-containing genes found in the genome) were identified in cultures grown on at least one of the substrates (Additional file 10). Eight and 61 of these genes, respectively, contained CBM and GT domains. The remaining 197 transcripts were classified into 47 GH, 6 CE, 10 AA and 3 PL families, respectively. Most transcripts were transcribed on all three carbon sources at varying levels.

Many fungal CAZymes involved in the degradation of biomass are secreted into the environment. The 40 most highly expressed genes during growth on wheat bran and xylan, predicted to contain both a secretion signal and one or more CAZy domains, are shown in Table 2 (for a complete list, see Additional file 10). A high percentage of these were found to encode putative enzymes involved in plant biomass deconstruction, such as the degradation of xylan or xyloglucan (GH2, 5, 10, 11, 16 and 74, CE5), β-glucan (GH5, 6, 7, 16, 17, 55, 72, 74 and 132), cellulose (AA9, GH6, GH74), starch (GH13) or pectin (PL1, PL3). The most highly expressed transcript of a secreted CAZyme on wheat bran, however, was the putative GH72 β-1,3-glucanosyltransglycosylase MalCi_235.14. Transcripts of this gene were also highly abundant during growth on xylan. GH72 enzymes are known to be involved in elongation and remodelling of the β-1,3-glucan of the fungal cell wall [53]. Thus, MalCi_235.14 is not likely to be involved in plant biomass degradation, but may be important for hyphal growth during an abundance of nutrients. Similarly, several putative chitinase-encoding genes (GH18) were also highly expressed on wheat bran and xylan. Chitinases play a role in many aspects of the fungal life cycle, including cell wall remodelling and degradation of exogenous chitin as a nutrient source [54].

**Table 2:** The 40 most highly expressed genes encoding putatively secreted CAZymes in *M. cinnamomea* FCH 10.5

Gene ID	fpkm^a^	Domain^b^	Putative function^c^
a) Wheat bran			
MalCi_235.14	3225	GH72	β1,3-glucanosyltransferase
MalCi_655.10	2516	GH2	β-galactosidase
MalCi_664.5	2102	CE5	Cutinase
MalCi_372.12	1816	GH17	β1,3-glucosidase
MalCi_266.5	1377	GH17	β1,3-glucosidase
MalCi_695.20	1325	GH16-CBM18	Chitinase
MalCi_179.14	1310	GH132	β-glucosidase
MalCi_724.6	1052	AA11	LPMO
MalCi_790.1	848	GH47	α1,2-mannosidase
MalCi_808.35	712	CE5	Cutinase
MalCi_670.57	658	GH71-GH71	*endo*-α1,3-glucanase
MalCi_652.31	589	GH132	β1,3-glucanase
MalCi_489.39	511	GH5	*endo*-β1,4-glucanase
MalCi_460.6	409	GH7	Cellobiohydrolase
MalCi_646.16	406	GH5	*endo*-β1,4-mannosidase
MalCi_757.11	393	GH16	*endo*-β1,3/1,4-glucanase
MalCi_655.13	259	GH13	α-amylase
MalCi_617.15	227	GH11	*endo*-β1,4-xylanase
MalCi_646.9	199	AA6	1,4-benzoquinone reductase
MalCi_780.39	197	GH6	Cellobiohydrolase
MalCi_624.16	191	GH16	Glucanase
MalCi_504.4	176	GH5	*endo*-β1,4-mannosidase
MalCi_739.16	172	AA9	LPMO
MalCi_769.3	172	AA11	LPMO
MalCi_091.4	170	GH20	β-hexosaminidase
MalCi_695.4	159	CE5	Cutinase
MalCi_618.6	147	PL1	Pectate lyase
MalCi_667.7	144	GH74-GH47	endo-β1,4-glucanase
MalCi_652.77	123	PL3	Pectate lyase
MalCi_435.17	122	GH18-CBM50-CBM50-CBM18	Chitinase
MalCi_700.10	110	GH65	α,α-trehalase
MalCi_528.12	103	GH18	Chitinase
MalCi_123.8	98	AA9	LPMO
MalCi_398.1	94	GH55-GH55	β1,3-glucosidase
MalCi_219.18	92	PL1	Pectate lyase
MalCi_177.6	88	AA1	Multicopper oxidase
MalCi_787.11	86	AA7	Glucooligosaccharide oxidase
MalCi_534.8	82	GH7	*endo*-β1,4-glucanase
MalCi_596.9	82	AA6	1,4-benzoquinone reductase
MalCi_675.5	77	CE5	Cutinase
b) Beechwood xylan			
MalCi_646.9	4912	AA6	1,4-benzoquinone reductase
MalCi_235.14	3648	GH72	β1,3-glucanosyltransferase
MalCi_617.15	3059	GH11	*endo*-β1,4-xylanase
MalCi_372.12	1951	GH17	β1,3-glucosidase
MalCi_266.5	1849	GH17	β1,3-glucosidase
MalCi_655.10	1667	GH2	β-galactosidase
MalCi_179.14	1368	GH132	β-glucosidase
MalCi_670.57	1092	GH71-GH71	*endo*-α1,3-glucanase
MalCi_790.1	943	GH47	α1,2-mannosidase
MalCi_724.6	734	AA11	LPMO
MalCi_695.20	663	GH16-CBM18	Chitinase
MalCi_769.3	624	AA11	LPMO
MalCi_435.17	425	GH18-CBM50-CBM50-CBM18	Chitinase
MalCi_757.11	419	GH16	*endo*-β1,3/1,4-glucanase
MalCi_652.31	223	GH132	β1,3-glucanase
MalCi_219.18	211	PL1	Pectate lyase
MalCi_618.6	188	PL1	Pectate lyase
MalCi_588.8	174	GH10-CBM1	*endo*-β1,4-xylanase
MalCi_652.77	170	PL3	Pectate lyase
MalCi_700.10	149	GH65	α,α-trehalase
MalCi_646.16	144	GH5	*endo*-β1,4-mannosidase
MalCi_596.9	134	AA6	1,4-benzoquinone reductase
MalCi_298.13	133	GH18	Chitinase
MalCi_398.1	129	GH55-GH55	β1,3-glucosidase
MalCi_808.35	128	CE5	Cutinase
MalCi_667.7	116	GH74-GH47	endo-β1,4-glucanase
MalCi_460.6	111	GH7	Cellobiohydrolase
MalCi_795.24	81	GH20	β-hexosaminidase
MalCi_666.7	81	GH24	Lysozyme
MalCi_091.4	73	GH20	β-hexosaminidase
MalCi_528.12	69	GH18	Chitinase
MalCi_695.4	68	CE5	Cutinase
MalCi_739.16	67	AA9	LPMO
MalCi_780.39	62	GH6	Cellobiohydrolase
MalCi_664.5	55	CE5	Cutinase
MalCi_682.37	54	GH2	*exo*-β-glucosaminidase
MalCi_177.6	53	AA1	Multicopper oxidase
MalCi_624.16	53	GH16	Glucanase
MalCi_381.4	49	GT31	Chondroitin sulphate synthase
MalCi_787.11	47	AA7	Glucooligosaccharide oxidase

^a^Normalised expression level of transcripts, average of two biological replicates

^b^CAZy domain as predicted by dbCAN 5.0

^c^Putative function deduced from first hit in BLASTp search against UniprotKB/Swiss-prot database

### Differential gene expression on wheat bran and xylan

**Fig. 4:**
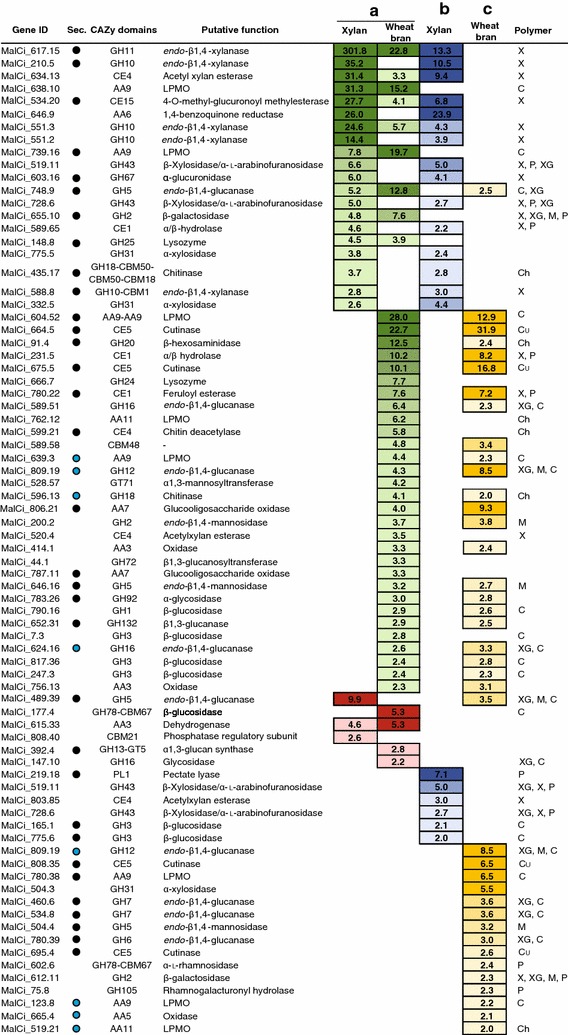
Differentially expressed CAZymes during cultivation on xylan and wheat bran. **a** Fold change of gene expression during cultivation on xylan and wheat bran, compared to glucose. Upregulated genes (i.e. transcripts more abundant on xylan and/or wheat bran) are shaded green, downregulated genes (i.e. transcripts more abundant on glucose) are shaded red. **b** Fold change of transcripts more abundant during growth on xylan than on wheat bran (blue shading). **c** Fold change of transcripts more abundant during growth on wheat bran than on xylan (yellow shading). Shading is stronger for higher fold changes for all cases. Transcripts were considered significantly differentially expressed when fold change was at least two (*p* ≤ 0.05). Sec., genes encoding putatively secreted proteins. The prediction was done with two algorithms, SignalP 4.1 (black circles signify proteins with recognised signal peptide), and WoLF PSORT (blue circles signify proteins predicted to be secreted)

To obtain information on how gene expression is influenced during growth on wheat bran and beechwood xylan, in comparison to glucose, a differential expression analysis was conducted. Using a log2 increase of at least 1 (i.e. a twofold enrichment) in relative transcript abundance as the significant threshold (adjusted *p* value < 0.05), 438 and 150 unique sequences were considered significantly more abundant in the samples grown on wheat bran and beechwood xylan, respectively, compared to glucose. Among the differentially expressed genes, many putative CAZymes that act on biopolymers, were found (Fig. 4, Additional file 11). When cultivated on wheat bran, the most highly upregulated transcript containing a CAZy domain (27.9 times more abundant than on glucose) stemmed from gene MalCi_604.52, comprising two predicted AA9 domains. Three other AA9 s were also highly upregulated (MalCi_739.16, MalCi_638.10, MalCi_639.3), as well as genes typically associated with cellulose degradation (GH1: MalCi_790.16; GH3: MalCi_7.3, MalCi_247.3, MalCi_817.36; GH5: MalCi_646.16, MalCi_748.9). Although fewer cellulose-active genes were upregulated in *M. cinnamomea* when grown on xylan, transcripts of two AA9 s (MalCi_780.38, MalCi_638.10) and one GH5 (MalCi_748.9) were still more abundant than when the fungus was cultivated on glucose (Fig. 4). Other genes encoding putative AA family members, apart from AA9 s, were also upregulated: a putative 1,4-benzoquinone reductase (AA6, MalCi_646.9), an AA11 LPMO (MalCi_762.12) and two AA3 oxidases (AA3, MalCi_414.1, MalCi_756.13), as well as two AA7 domain-containing genes (MalCi_787.11, MalCi_806.21).

Several genes encoding putative hemicellulose-active enzymes were upregulated after cultivation on both wheat bran and xylan; the most highly upregulated gene encoding a GH11 putative *endo*-β1,4-xylanase (MalCi_617.15) (Fig. 4). Its transcripts were 301.8 times more abundant during growth on xylan and 22.79 more abundant during growth on wheat bran, respectively, compared to glucose. Four additional putative *endo*-β1,4-xylanases were more abundant on xylan, compared to glucose: MalCi_210.5 (GH10), MalCi_551.3 (GH10), MalCi_551.2 (GH10) and MalCi_588.8 (GH10-CBM1). Of these, only MalCi_551.3 was also significantly upregulated during growth on wheat bran. The expression of two putative CE5 genes (encoding acetyl xylan esterases or cutinases) was strongly increased during growth on wheat bran with MalCi_664.5 and MalCi_675.5 being 23.7 times and 10.1 times, respectively, more abundant on wheat bran than on glucose. Furthermore, the following genes, which encode accessory xylanolytic enzymes that liberate side groups from the main chain, were differently expressed during cultivation on wheat bran and xylan, compared to glucose: three CE4 putative acetyl xylan esterases (MalCi_634.13, MalCi_599.21, MalCi_520.4); a GH43 putative β-xylosidase (MalCi_519.11) and a GH43 putative β-xylosidase or α-l-arabinofuranosidase (MalCi_728.6), which remove xylose or arabinose units from the non-reducing end; a GH67 putative α-glucuronidase (MalCi_603.16), which cleaves glucuronic acid side groups from xylans; and two GH2 putative β-galactosidases (MalCi_655.10, MalCi_200.2).

Hemicelluloses are crosslinked to lignin via ferulic acid or glucuronic acid ester linkages [55]. Ferulic acid esters are particularly abundant in grasses, and accordingly, a gene encoding a putative feruloyl esterase (CE1, MalCi_780.22) was upregulated 7.6 times on wheat bran. Transcripts of a putative glucuronoyl esterase (CE15, MalCi_534.2), which is thought to hydrolyse the ester linkages between lignin and 4-*O*-methyl-d-glucuronic acid side chains of xylan [56], were more abundant on both wheat bran (4.1 times) and xylan (27.7 times) than on glucose. The expression of several genes with predicted secretion signals but lacking known function by BLAST were upregulated on either wheat bran, xylan or both. MalCi_132.2 (xylan: 15.1-fold; wheat bran: 62.2-fold), MalCi_565.14 (xylan: 7.6-fold; wheat bran: 10.5-fold), MalCi_610.8 (xylan: 3.3-fold; wheat bran: 8.8-fold), MalCi_521.23 (wheat bran: 16.4-fold), MalCi_666.6 (wheat bran: 7.0-fold) and MalCi_398.7 (wheat bran: 4.3-fold). In addition, one gene comprising a CBM9 as the only identified domain was 28.5-fold more abundant on wheat bran, compared to glucose. Of the 87 genes, whose transcripts were considered significantly more abundant in *M. cinnamomea* after cultivation on both xylan and wheat bran, nine comprised predicted CAZy domains and eight of those are likely involved in biopolymer deconstruction (Fig. 4). Many genes were also differently expressed during growth on wheat bran compared to growth on xylan. In total, 355 transcripts were more abundant on xylan, and 410 more abundant on wheat bran (adjusted *p* value < 0.05, log2 ≥ 1). Among these, 20 transcripts containing CAZy domains were upregulated during growth on xylan and 39 on wheat bran (Fig. 4, Additional file 11). The genes more abundantly expressed on xylan included many putative xylanases of the GH10 and GH11 families, while a wider variety of putative CAZymes was upregulated after growth on wheat bran, primarily AA9, GH1, 3 and 5 family members, as well as putative feruloyl esterases, acetylxylan esterases and cutinases of the CE1, 4 and 5 families, respectively. A gene set analysis of the genes found to be upregulated during growth of *M. cinnamomea* on wheat bran and xylan revealed that the following GO categories were most enriched: GO:0016798 (hydrolase activity, acting on glycosyl bonds), GO:0016491 (oxidoreductase activity), GO:0022857 (transmembrane transporter activity), GO:0005975 (carbohydrate metabolic process) and GO:0006629 (lipid metabolic process) (Additional file 12).

Far fewer genes were downregulated after growth on wheat bran (195) and xylan (34) compared to glucose (Additional file 11). Among the downregulated genes on wheat bran, four were identified that comprise CAZy domains: a gene encoding a GH16 putative glycosidase (MalCi_147.10), a GT5-GH13 multimodular putative cell wall α-1,3-glucan synthase (MalCi_392.4), a GH78-CBM67 putative β-glucosidase (MalCi_177.4) and an AA3 family protein (MalCi_615.33). On xylan, three putative CAZyme genes were downregulated: a gene comprising a CBM21 domain (MalCi_808.40), a gene encoding a GH5 putative β-1,4-glucanase (MalCi_489.39) and the same gene encoding a AA3 protein that was also downregulated on wheat bran.

Transcriptional regulators, like XlnR from *A. niger* and CreA from *A. nidulans*, are known to be involved in fungal biomass turnover [57]. Two orthologues of XlnR, MalCi_741.50 and MalCi_792.36, were both expressed in *M. cinnamomea* FCH 10.5 at similar levels when it was grown on glucose, wheat bran or xylan (Additional file 11). Similarly, the expression of two genes encoding orthologues of the repressor CreA, MalCi_342.1 and MalCi_11.1, was not significantly different on the three substrates tested.

## Discussion

In the present study, we have systematically explored the lignocellulolytic potential of the thermophilic fungus *M.* *cinnamomea* on a genomic level. The obtained information serves as a resource for detailed exploitation of its enzymes. We have performed a genomic analysis of the *M.* *cinnamomea*, strain FCH 10.5, an analysis of its transcriptional response when grown on beechwood xylan, wheat bran and glucose, and correlated the presence of putative CAZymes in the genome to its ability to grow on different carbohydrates. Our study shows that *M.* *cinnamomea* has an abundant repertoire of CAZy domain-containing genes that are distributed over 108 different CAZy families, and has furthermore the genetic potential to degrade the majority of naturally occurring plant cell wall polysaccharides. *M.* *cinnamomea* (syn. *M. pulchella* var. *sulfurea*) has been isolated from soil, decaying vegetation and self-heating hay stacks all over the world [58], suggesting that the species is widely distributed in the environment, and that it has a significant role in plant biomass degradation. Several enzymes from *M.* *cinnamomea* that are targeting biopolymers have been studied and expressed in recent years [18–25; Additional file 1], and all of these enzymes were reported to have temperature optima between 45 and 80 °C.

In a phylogenetic analysis including several well-studied biomass degraders, *M.* *cinnamomea* was found to be evolutionary closer to *Aspergillus* and *Penicillium* species (order Eurotiales) than *Myceliophthora* or *Neurospora* species (order Sordariales) [17]. However, its repertoire of CAZymes is much more similar to the phylogenetically more distant species *M.* *thermophila* and *N.* *crassa* (Table 1). Closely related fungal species were shown to use different sets of enzymes to hydrolyse the same complex biomass [59], while species with considerable evolutionary distance can display a similar arsenal of CAZymes. A strong influence of the natural habitat on the capability of fungi to degrade biomass, i.e. their repertoire of specific enzymatic activities, could be a possible explanation. This hypothesis is supported by the findings that the thermophilic *M. thermophila*, which is, like *M. cinnamomea*, commonly found in compost, hay stacks and sun-heated soil [60], has a CAZyme repertoire more similar to that of *M. cinnamomea* than the phylogenetically closer mesophiles *A. oryzae*, *A. niger*, *A. nidulans* and *P. chrysogenum* (Additional files 8, 9).

The growth profile data as well as the measured enzyme activities were generally in good agreement with the repertoire of putative CAZymes found in the *M. cinnamomea* genome as well as in the transcriptome analysis. The preferred substrates of *M. cinnamomea* FCH 10.5 among the tested ones were beechwood xylan, cellobiose and starch (Fig. 1c), which correlated well with the presence of related CAZymes in the genome (Additional file 8). Good growth could also be observed on inulin, even though no putative invertase or inulinase genes were identified in the genome, similar to what has been observed in *R. oryzae* [48]. *M. cinnamomea* also grew well on locust bean gum (galactomannan with galactose residues on every fourth mannose), despite the apparent absence of genes encoding α-1,6-galactosidases in its genome. As expected from the lack of genes encoding GH28 proteins, no growth was detected on citrus pectin as the sole carbon source. This indicates that the presence of only pectin-degrading lyases (PL1, PL3, PL4) in the genome of *M. cinnamomea* is not sufficient for the deconstruction of this polymer. The Ascomycete *Podospora anserina* also completely lacks family GH28 proteins and has similarly been found to grow very poorly on pectin substrates [61]. Surprisingly, only poor growth of *M. cinnamomea* FCH 10.5 was observed on cellulose (Avicel, CMC), despite the presence of a large number of genes encoding putative cellulose-degrading enzymes in its genome.

The induction of many CAZy genes upon transfer to cellulose- or hemicellulose-containing media happens within a few hours in *A. fumigatus* [62]. In *M. cinnamomea* FCH 10.5, the transcriptional response to growth on xylan and wheat bran after 4 and 48 h of cultivation was clearly distinct from that on glucose; about 18% of all GH genes in the genome were being upregulated more than twofold. Most CAZy domain-containing genes with significantly increased expression when grown on wheat bran or xylan were predicted to target cellulose and hemicelluloses (xylan, xyloglucan, galacto(gluco)mannan) (Fig. 4). In particular, putative GH10 and GH11 *endo*-β1,4-xylanases, GH43 β1,4-xylosidases, CE5 acetyl xylan esterases and cutinases, a CE15 4-*O*-methyl-glucuronoyl methylesterase and a CE1 feruloyl esterase, as well as AA9 LPMOs, were more abundant on either xylan and wheat bran, or both. The bran fraction of wheat consists of about 16% proteins and 80% carbohydrates, the rest being mostly cutin, lignin, uronic acids, polyphenols, ferulic acid, phytic acid and minerals [63]. The starch content of wheat bran is about 10%, and of the non-starch polysaccharides, arabinoxylans are the most prevalent (70%), followed by cellulose (24%) and β-glucan (6%) [64]. The more complex composition of wheat bran was reflected in the upregulation of a more diverse set of CAZymes in *M. cinnamomea* than during growth on beechwood xylan, which is a comparatively simple substrate. Several genes that are not directly involved in the decomposition of xylan, such as AA9 LPMOs, which usually act on cellulose and xyloglucan, were nevertheless upregulated during growth on beechwood xylan. This shows that both xylan as well as more complex biomass (like wheat bran) can individually induce the expression of gene families generally known to be involved in cellulose and hemicellulose turnover. The expression of hemicellulose-degrading, but not cellulose-degrading, genes has been observed in *Fusarium* *graminearum* and *N.* *crassa* when grown on xylan [65–67], while the concomitant induction of the cellulolytic system by XlnR has been reported for other fungi [62]. The poor growth of *M. cinnamomea* FCH 10.5 on cellulose (Avicel or CMC) was surprising (Fig. 1c), since its genome encodes many putative cellulolytic enzymes, including putative AA9 LPMOs. It needs to be investigated further if these AA9 proteins truly act on cellulose, or if they have activity on other polymers.

Wheat bran contains a considerable fraction of the waxy polymer cutin [68], and consequently we found several CE5 putative cutinases to be upregulated in *M. cinnamomea* when grown on wheat bran, one of which (MalCi_664.5) has recently been cloned and characterised [25]. Genes encoding CAZymes not involved in plant cell wall degradation were also found among the differentially expressed genes, e.g. two GH18 putative chitinases genes, and two genes with AA7 domains (gluco- or chitooligosaccharide oxidases), possibly involved in the reorganisation of the fungal cell wall.

Of the CBMs targeting different polymers, cellulose-binding CBM1s are particularly abundant in Ascomycetes and white-rot Basidiomycetes, with on average over 30% of CBMs belonging to family 1 [52]. The CBM1 abundance was unusually low in *M.* *cinnamomea*; only one member was found in a gene encoding a GH10-CBM1 multimodular protein (MalCi_588.8). CBMs typically increase enzyme–substrate interaction, but low water content of the substrate has been shown to lead to equal hydrolytic performance of a cellulase with and without a CBM1, by enhancing the enzyme–substrate interaction irrespectively of the presence of a CBM1 [51, 69]. The low number of CBMs, particularly CBM1s, in the genome of *M.* *cinnamomea* might, therefore, be a consequence of its occurrence in rather dry environments, such as compost, hay stacks and soil. We also discovered genes containing CBM domain(s), but lacking catalytic domain(s), which may point to yet unclassified functions and potentially new CAZy families. Eight genes with those structures were identified in the genome of FCH 10.5 (Fig. 3). For gene Malci_300.14, dbCAN predicted a single cellulose-binding CBM46 domain (CBM_X2 in CDD search), which is a family found in GHs and scaffolding proteins of cellulosomes, and for which binding to bacterial cell walls and cellulose has been demonstrated [70]. Genes MalCi_332.1, MalCi_557.32 and MalCi_799.34 contain one or several CBM50 (CDD: LysM) domains and MalCi_326.2 a CBM18 domain, both of which can be part of chitinases and enzymes involved in bacterial cell wall degradation [71, 72]. A CBM48 domain was found in gene MalCi_589.58, which often has a glycogen-binding function, but can also be associated with starch-degrading enzymes, such as pullulanases or isoamylases [73]. A truncated predicted cellulose-binding CBM10 domain was identified in gene MalCi_664.9, a predicted starch-binding CBM21 domain in gene MalCi_808.4 and a predicted cellulose- or glucomannan-binding CBM16 domain in gene MalCi_427.10. Further studies are needed to determine the functions of these proteins.

A recent proteomic analysis of an *M.* *cinnamomea* strain cultivated on sorghum straw found a similar repertoire of secreted CAZymes to those inferred in the present study from the expression of CAZy domain-containing genes with predicted secretion signals (Table 2, Additional file 10), including many enzymes involved in the degradation of cellulose, hemicelluloses and chitin, as well as proteases and oxidases [26]. Interestingly, no AA9 family protein was identified in the secretome, although we found seven putative LPMOs with AA9 domains in the genome, four of which were upregulated on wheat bran and/or xylan, and one of which was among the 30 most highly expressed secreted CAZymes during growth on wheat bran. However, an AA8 family protein was found in the secretome [26], which can serve as an electron donor in the reduction of Cu(II) to Cu(I) in LPMOs [74]. We confirmed the presence of a single AA8 domain-containing gene (MalCi_654.4) in the *M.* *cinnamomea* genome, transcribed during cultivation on all substrates, though not significantly differentially expressed. A gene containing a GH10 and a CBM1 domain was the only candidate we identified with a CBM1 domain, whereas Mahajan et al. found three: a GH10-CBM1, a CE15-CBM1 and a GH6-CBM1 [26]. Neither the CE15 nor the GH6 domain was found to be associated with a CBM domain in the present study. All of the four GH10 genes identified in the genome of *M. cinnamomea* in this study (MalCi_210.5, MalCi_551.2, MalCi_551.3, MalCi_588.8) were expressed during growth on xylan, as well as during growth on wheat bran albeit to a much lower level (Additional file 10). Wheat bran is more similar in composition to the sorghum straw used by Mahajan et al. which may explain the detection of only three GH10 proteins in the secretome [26].

## Conclusions

The findings of this study showcase the large repertoire of genes encoding CAZy domain-containing proteins in the genome of *M.* *cinnamomea* FCH 10.5, a fungus which grows at high temperatures (> 50 °C) and is an efficient producer of many plant cell wall degrading enzymes. Genome sequencing and transcriptomics enabled the identification of genes actively involved in the degradation of xylan and wheat bran. The presented data enhance our understanding of fungal biodiversity, with special regard to differences in the mechanisms of plant cell wall degradation, and will facilitate the use of thermophilic enzymes produced by *M. cinnamomea* in biorefinery applications.

## Additional files

**Additional file 1.** Comparison of characterised enzymes from *M. cinnamomea* from the literature, and their corresponding gene model from *M. cinnamomea* FCH 10.5.

**Additional file 2.** Scatter plots of the biological replicates of the RNAseq data.

**Additional file 3.** Multiple and pairwise sequence alignments of *M. cinnamomea* ITS1-ITS2-5.8S-rRNA sequences deposited at Genbank.

**Additional file 4.** Xylanase and endoglucanase (CMCase) activities.

**Additional file 5.** Growth of *M. cinnamomea* FCH 10.5 and four other filamentous fungi on mono- and polysaccharides.

**Additional file 6.** Genome statistics, BUSCO analysis output and BLAST hits of *M. cinnamomea* FCH 10.5 genome assembly.

**Additional file 7.** GO classification of annotated proteins in *M. cinnamomea* FCH 10.5.

**Additional file 8.** Comparative analysis of the number of CAZy families (GHs, GTs, CEs, PLs, AAs, CBMs) in 24 different fungi.

**Additional file 9.** Phylogenetic tree of ITS1-ITS2-5.8S rRNA sequences.

**Additional file 10.** Average fpkm values of CAZy-domain containing transcripts expressed during growth on beechwood xylan, wheat bran and glucose.

**Additional file 11.** Differentially expressed genes of *M. cinnamomea* FCH 10.5 cultivated on wheat bran, beechwood xylan or glucose.

**Additional file 12.** GO enrichment of upregulated genes during cultivation on wheat bran or beechwood xylan, compared to glucose.
