# Supplementary material for: Combined genome and transcriptome sequencing to investigate the plant cell wall degrading enzyme system in the thermophilic fungus Malbranchea cinnamomea
Source: Biotechnol Biofuels. 2017 Nov 13;10:265. doi: 10.1186/s13068-017-0956-0 (PMC5683368; doi:10.1186/s13068-017-0956-0)

**Additional File S2**

**Scatterplot of the biological duplicates of the RNAseq data.**

Data points are normalised read counts with each point representing an expressed gene. Samples are RNA from *M. cinnamomea* FCH 10.5 grown on glucose (Glucose Replicate 1, 2), wheat bran (Wheat bran Replicate 1, 2) or beechwood xylan (Xylan Replicate 1, 2).


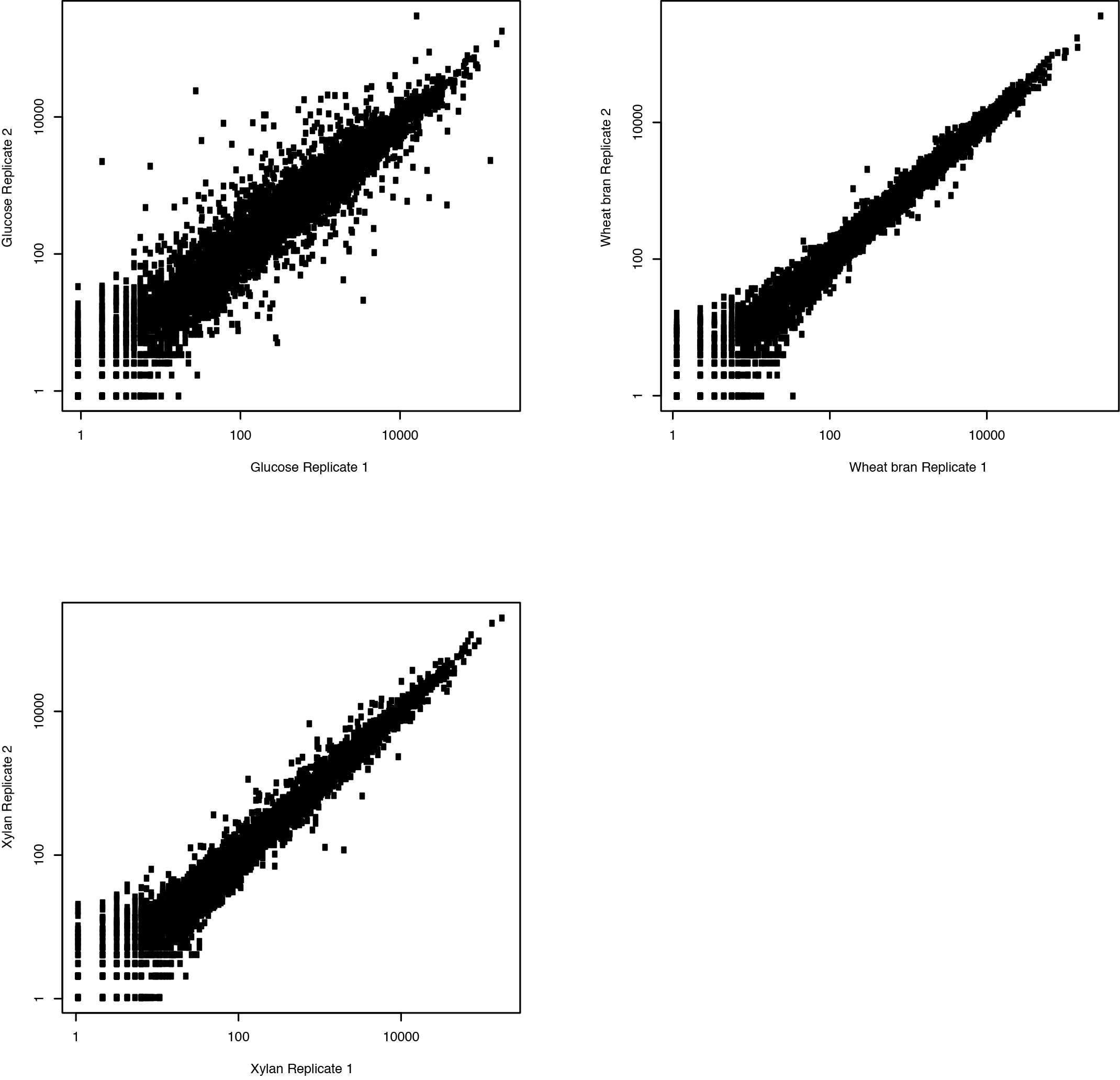

Supplement: Supplementary file 2 — Additional file 2. Scatter plots of the biological replicates of the RNAseq data. [file 13068_2017_956_MOESM2_ESM.docx]
