# Supplementary material for: Combined genome and transcriptome sequencing to investigate the plant cell wall degrading enzyme system in the thermophilic fungus Malbranchea cinnamomea
Source: Biotechnol Biofuels. 2017 Nov 13;10:265. doi: 10.1186/s13068-017-0956-0 (PMC5683368; doi:10.1186/s13068-017-0956-0)
Supplement: Supplementary file 3 — Additional file 3. Multiple and pairwise sequence alignments of M. cinnamomea ITS1-ITS2-5.8S-rRNA sequences deposited at Genbank. [file 13068_2017_956_MOESM3_ESM.docx]

**Additional File S3**

**Multiple and pairwise sequence alignments of *Malbranchea cinnamomea* ITS1-ITS2-5.8S rRNA sequences deposited at Genbank.**

Genbank was accessed on 5 September 2017 to retrieve the ITS sequences, which were clipped so that all sequences cover the same length. Multiple sequence alignment was done with MUSCLE 3.8 and pairwise sequence alignments were done with EMBOSS Needle [1].

Sequences used

| \| **MF838862** \| \| --- \| | FCH 10.5, isolated from compost at waste treatment facility in Hanoi; present study |
| --- | --- | --- |
| **KJ834332.1** | Z1E-1, isolated from wheat straw during mushroom compost production; [2] |
| **JF412018.2** | CBS 343.55; isolated in the Netherlands; [3] |
| **KY992588.1** | Gz1, isolated from spent mushroom substrate, China |
| **KT279416.1** | GSMBKU, isolated in India |
| **KJ834368.1** | Z1C-52, isolated from wheat straw during mushroom compost production; [2] |
| **KJ563258.1** | CM-10T, isolated from composting soil near Punjab, semi-arid Amritsar zone; [4] |
| **JF922020.1** | CBS 960.72, isolated in France; [5] |
| **KJ834339.1** | Z1E-9, isolated from wheat straw during mushroom compost production; [2] |

CLUSTAL multiple sequence alignment by MUSCLE (3.8)

JF922020.1 TGCGATTAAGTATGTGAATTGCAGAATTCCGTGAATCATCGAATCTTTGAACGCACATGG

KJ563258.1 TGCGATAAGT-ATGTGAATTGCAGAATTCCGTGAATCATCGAATCTTTGAACGCACATGG

KJ834368.1 TGCGATAAGTAATGTGAATTGCAGAATTCCGTGAATCATCGAATCTTTGAACGCACATGG

KJ834339.1 TGCGATAAGTAATGTGAATTGCAGAATTCCGTGAATCATCGAATCTTTGAACGCACACGG

KT279416.1 TGCGATAAGTAATGTGAATTGCAGAATTCCGTGAATCATCGAATCTTTGAACGCACCTGG

FCH_10.5 TGCGATAAGTAATGTGAATTGCAGAATTCCGTGAATCATCGAATCTTTGAACGCACATGG

KJ834332.1 TGCGATAAGTAATGTGAATTGCAGAATTCCGTGAATCATCGAATCTTTGAACGCACATGG

KY992588.1 TGCGATAAGTAATGTGAATTGCAGAATTCCGTGAATCATCGAATCTTTGAACGCACATGG

JF412018.2 TGCGATAAGT-ATGTGAATTGCAGAATTCCGTGAATCATCGAATCTTTGAACGCACATGG

****** * ********************************************* **

JF922020.1 CGCCCTCTGGTATTCCGGGGGGCATGCCTGTCCGAGCGTCATTGCAACCCTCAAGCGCGG

KJ563258.1 CGCCCTCTGGTATTCCGGGGGGCATGCCTGTCCGAGCGTCATTGCAACCCTCAAGCGCGG

KJ834368.1 CGCCCTCTGGTATTCCGGGGGGCATGCCTGTCCGAGCGTCATTGCAACCCTCAAGCGCGG

KJ834339.1 CGCCCTCTGGTATTCCGGGGGGCATGCCTGTCCGAGCGTCATTGCAACCCTCAAGCGCGG

KT279416.1 CGCCCTCTGGTATTCCGGGGGGCATGCCTGTCCGAGCGTCATTGCAACCCTCAAGCGCGG

FCH_10.5 CGCCCTCTGGTATTCCGGGGGGCATGCCTGTCCGAGCGTCATTGCAACCCTCAAGCGCGG

KJ834332.1 CGCCCTCTGGTATTCCGGGGGGCATGCCTGTCCGAGCGTCATTGCAACCCTCAAGCGCGG

KY992588.1 CGCCCTCTGGTATTCCGGGGGGCATGCCTGTCCGAGCGTCATTGCAACCCTCAAGCGCGG

JF412018.2 CGCCCTCTGGTATTCCGGGGGGCATGCCTGTCCGAGCGTCATTGCAACCCTCAAGCGCGG

************************************************************

JF922020.1 CTTGTGTGATGGGCGCGCGT-CCCCCCGAACTGGGCGGGACGGGCCCGAAAGGCAGTGGC

KJ563258.1 CTTGTGTGATGGGCGCGCGTCCCCCCCGAACTGGGCGGGACGGGCCCGAAA-GCAGTGGC

KJ834368.1 CTTGTGTGATGGGCGCGCGT-CCCCCCGAACTGGGCGGGACGGGCCCGAAAGGCAGTGGC

KJ834339.1 CTTGTGTGATGGGCGCGCGTCCCCCCCGAACTGGGCGGGACGGGCCCGAAAGGCAGTGGC

KT279416.1 CTTGTGTGATGGGCGCGCGTCCCCCCCGAACTGGGCGGGACGGGCCCGAAAGGCAGTGGC

FCH_10.5 CTTGTGTGATGGGCGCGCGTCCCCCCCGAACTGGGCGGGACGGGCCCGAAAGGCAGTGGC

KJ834332.1 CTTGTGTGATGGGCGCGCGTCCCCCCCGAACTGGGCGGGACGGGCCCGAAAGGCAGTGGC

KY992588.1 CTTGTGTGATGGGCGCGCGTCCCCCCCGAACTGGGCGGGACGGGCCCGAAAGGCAGTGGC

JF412018.2 CTTGTGTGATGGGCGCGCGT-CCCCCCGAACTGGGCGGGACGGGCCCGAAAGGCAGTGGC

******************** ****************************** ********

JF922020.1 GGCGTCGCGGTCATCCTCGATCGCCCGAGTGCATGGGGCTCAGTCACGCACGACGGCGAG

KJ563258.1 GGCGTCG-GGTCATCCTCGATCGCGCGAGTGCATGGGGCTCAGTCACGCACGACGGCGAG

KJ834368.1 GGCGTCGCGGTCATCCTCGATCGCCCGAGTGCGTGGGGCTCAGTCACGCACGACGGCGAG

KJ834339.1 GGCGTCGCGGTCATCCTCGATCGCCCGAGTGCATGGGGCTCAGTCACGCACGACGGCGAG

KT279416.1 GGCGTCGCGGTCATCCTCGATCGCCCGAGTGCATGGGGCTCAGTCACGCACGACGGCGAG

FCH_10.5 GGCGTCGCGGTCATCCTCGATCGCCCGAGTGCATGGGGCTCAGTCACGCACGACGGCGAG

KJ834332.1 GGCGTCGCGGTCATCCTCGATCGCCCGAGTGCATGGGGCTCAGTCACGCACGACGGCGAG

KY992588.1 GGCGTCGCGGTCATCCTCGATCGCCCGAGTGCATGGGGCTCAGTCACGCACGACGGCGAG

JF412018.2 GGCGTCGCGGTCATCCTCGATCGCCCGAGTGCATGGGGCTCAGTCACGCACGACGGCGAG

******* **************** ******* ***************************

JF922020.1 GACCGGCGCCGGCCG

KJ563258.1 GACCGGCGCCGGCCG

KJ834368.1 GACCGGCGCCGGCCG

KJ834339.1 GACCGGCGCCGGCCG

KT279416.1 GACCGGCGCCGGCCG

FCH_10.5 GACCGGCGCCGGCCG

KJ834332.1 GACCGGCGCCGGCCG

KY992588.1 GACCGGCGCCGGCCG

JF412018.2 GACCGGCGCCGGCCG

***************

Percent identity of pairwise sequence alignments of clipped ITS sequences

|  | **FCH 10.5** | **KJ834332.1** | **JF412018.2** | **KY992588.1** | **KT279416.1** | **KJ834368.1** | **KJ563258.1** | **JF922020.1** | **KJ834339.1** |
| --- | --- | --- | --- | --- | --- | --- | --- | --- | --- |
| **FCH 10.5** | **100.0** |  |  |  |  |  |  |  |  |
| **KJ834332.1** | **100.0** | **100.0** |  |  |  |  |  |  |  |
| **JF412018.2** | 99.2 | 99.2 | **100.0** |  |  |  |  |  |  |
| **KY992588.1** | **100.0** | **100.0** | 99.2 | **100.0** |  |  |  |  |  |
| **KT279416.1** | 99.6 | 99.6 | 98.8 | 99.6 | **100.0** |  |  |  |  |
| **KJ834368.1** | 99.2 | 99.2 | 99.2 | 99.6 | 98.8 | **100.0** |  |  |  |
| **KJ563258.1** | 98.4 | 98.4 | 98.4 | 98.4 | 98.0 | 97.6 | **100.0** |  |  |
| **JF922020.1** | 98.6 | 98.8 | 99.6 | 98.8 | 98.4 | 98.8 | 98.0 | **100.0** |  |
| **KJ834339.1** | 99.6 | 99.6 | 98.8 | 99.6 | 99.2 | 98.8 | 98.0 | 98.4 | **100** |

**References**

1. http://www.ebi.ac.uk. Accessed 5 September 2017.

2. Zhang X, Zhong Y, Yang S, Zhang W, Xu M, Ma A, et al. Diversity and dynamics of the microbial community on decomposing wheat straw during mushroom compost production. Bioresour. Technol. 2014;170:183–95.

3. Morgenstern I, Powlowski J, Ishmael N, Darmond C, Marqueteau S, Moisan MC, et al. A molecular phylogeny of thermophilic fungi. Fungal Biol. 2012;116:489–502.

4. Mahajan C, Chadha BS, Nain L, Kaur A. Evaluation of glycosyl hydrolases from thermophilic fungi for their potential in bioconversion of alkali and biologically treated *Parthenium hysterophorus* weed and rice straw into ethanol. Bioresour. Technol. 2014;163:300–7.

5. Pettersson OV, Leong SLL, Lantz H, Rice T, Dijksterhuis J, Houbraken J, et al. Phylogeny and intraspecific variation of the extreme xerophile, *Xeromyces bisporus*. Fungal Biol. 2011;115:1100–11.
