# Supplementary material for: Combined genome and transcriptome sequencing to investigate the plant cell wall degrading enzyme system in the thermophilic fungus Malbranchea cinnamomea
Source: Biotechnol Biofuels. 2017 Nov 13;10:265. doi: 10.1186/s13068-017-0956-0 (PMC5683368; doi:10.1186/s13068-017-0956-0)
Supplement: Supplementary file 4 — Additional file 4. Xylanase and endoglucanase (CMCase) activities. [file 13068_2017_956_MOESM4_ESM.docx]

**Additional File S4**

**Xylanase and endoglucanase activities**


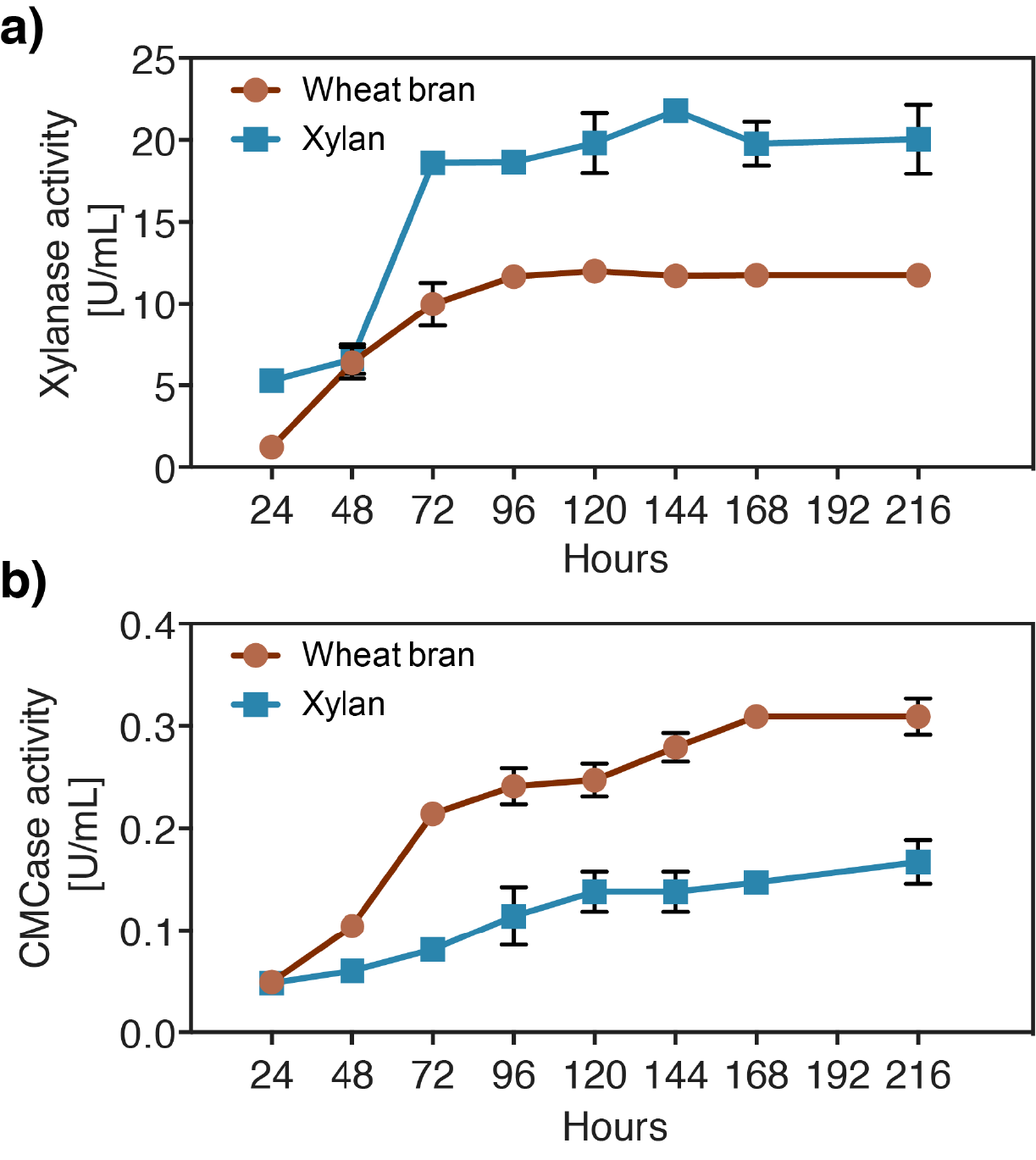


**Additional Files S4**. Enzyme assays evaluating xylanase (a) and endoglucanase (CMCase) (b) activities in the culture filtrate of M. cinnamomea FCH 10.5 cultivations on beechwood xylan (blue) and wheat bran (red) over the course 216 hours (=9 days).
