# Supplementary material for: Combined genome and transcriptome sequencing to investigate the plant cell wall degrading enzyme system in the thermophilic fungus Malbranchea cinnamomea
Source: Biotechnol Biofuels. 2017 Nov 13;10:265. doi: 10.1186/s13068-017-0956-0 (PMC5683368; doi:10.1186/s13068-017-0956-0)
Supplement: Supplementary file 5 — Additional file 5. Growth of M. cinnamomea FCH 10.5 and four other filamentous fungi on mono- and polysaccharides. [file 13068_2017_956_MOESM5_ESM.docx]

**Additional File S5**

**Growth of *M. cinnamomea* FCH 10.5 and four other filamentous fungi on mono- and polysaccharides.**


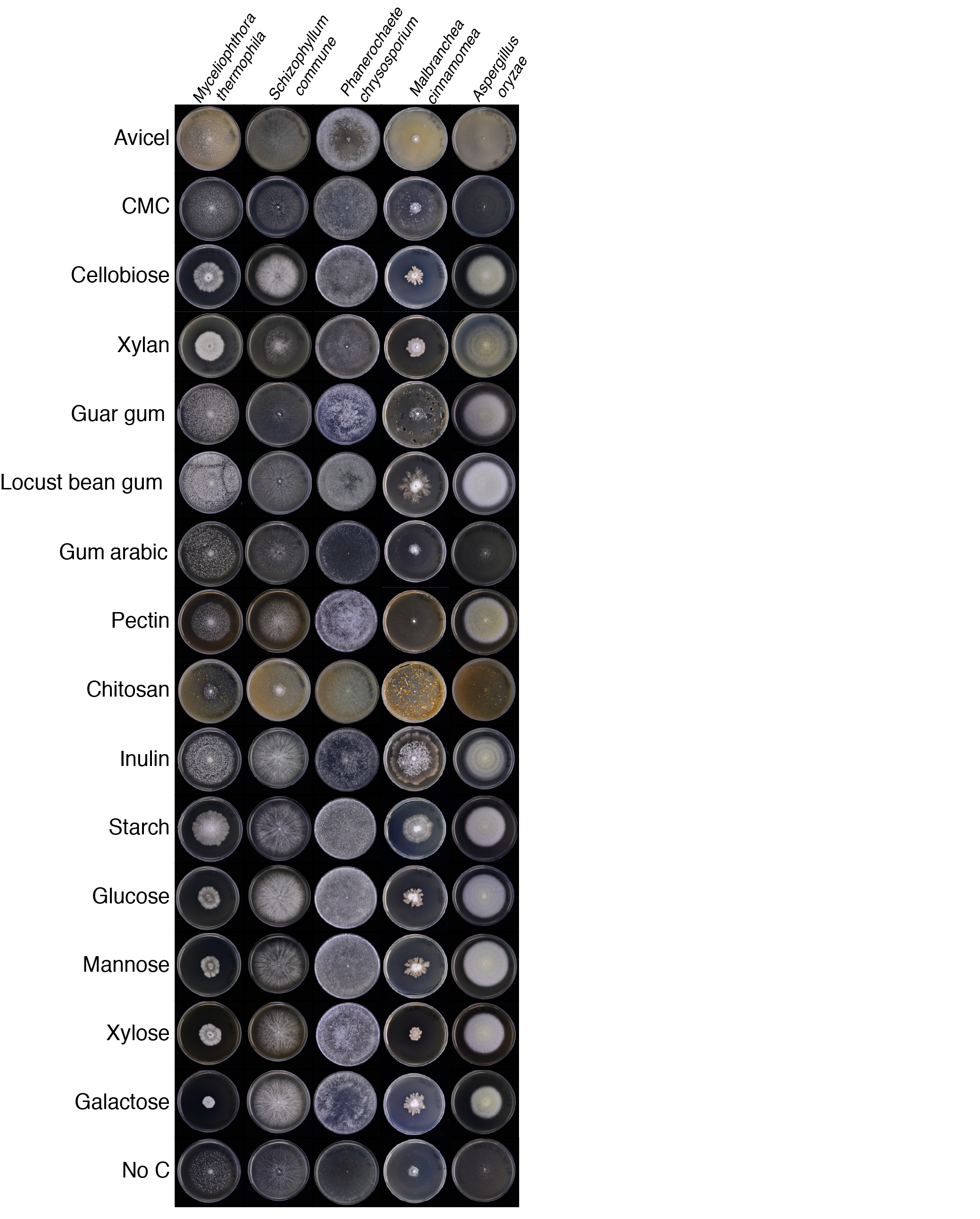


**Additional Files S5**. Growth of *M. cinnamomea* FCH 10.5 and four other filamentous fungi on agar media containing cellulose (Avicel, carboxymethyl cellulose (CMC), cellobiose, plant polysaccharides (beechwood xylan, guar gum, locust bean gum, gum arabic, pectin, inulin, starch), chitosan, monosaccharides (glucose, mannose, xylose, galactose) and no carbon (no C).
