# Supplementary material for: Combined genome and transcriptome sequencing to investigate the plant cell wall degrading enzyme system in the thermophilic fungus Malbranchea cinnamomea
Source: Biotechnol Biofuels. 2017 Nov 13;10:265. doi: 10.1186/s13068-017-0956-0 (PMC5683368; doi:10.1186/s13068-017-0956-0)
Supplement: Supplementary file 7 — Additional file 7. GO classification of annotated proteins in M. cinnamomea FCH 10.5. [file 13068_2017_956_MOESM7_ESM.docx]

**Additional File S7**

**GO classification of annotated proteins in *M. cinnamomea* FCH 10.5.**
