# Supplementary material for: Combined genome and transcriptome sequencing to investigate the plant cell wall degrading enzyme system in the thermophilic fungus Malbranchea cinnamomea
Source: Biotechnol Biofuels. 2017 Nov 13;10:265. doi: 10.1186/s13068-017-0956-0 (PMC5683368; doi:10.1186/s13068-017-0956-0)
Supplement: Supplementary file 9 — Additional file 9. Phylogenetic tree of ITS1-ITS2-5.8S rRNA sequences. [file 13068_2017_956_MOESM9_ESM.docx]

**Additional File S9**

**Phylogenetic tree of ITS1-ITS2-5.8S rRNA sequences**

ITS1-ITS2-5.8S rRNA sequence of *Malbranchea cinnamomea* FCH 10.5, amplified with primers ITS1 and ITS4, and ITS1-ITS2-5.8S rRNA sequences of 15 Ascomycetes, six Basidiomycetes, one Zygomycete and one Oomycete used for the generation of the phylogenetic tree in Figure 1c.

Ascomycetes: *Yarrowia lipolytica, Saccharomyces cerevisiae, Klyveromyces lactis, Arthrobotrys oligospora, Penicillium chrysogenum, Aspergillus nidulans, Aspergillus niger, Aspergillus oryzae, Leptosphaeria maculans, Malbranchea cinnamomea, Thielavia terrestris, Myceliophthora thermophila, Podospora anserina, Neurospora crassa, Magnaporthe grisea, Fusarium graminearum*

Basidiomycetes: *Rhodosporidium toruloides, Sporisorium reilianum, Schizophyllum commune, Piriformospora indica, Phanerochaete chrysosporium, Postia placenta,*

Zygomycete: *Rhizopus oryzae*

Oomycete: *Phytophthora infestans*

**Materials and Methods**

*M. cinnamomea* FCH 10.5 DNA was used to amplify the internal transcribed spacer (ITS) region with the universal primers ITS1 (forward; 5’ TCCGTAGGTGAACCTGCGG 3’) and ITS4 (reverse; 5’ TCCTCCGCTTATTGATATGC 3’) [1]. Amplicons were sequenced and compared to available databases using BLAST. A phylogenetic tree, based on aligned amplified ITS1-ITS2-5.8S rRNA sequences of 16 Ascomycetes, six Basidiomycetes, one Zygomycete and one Oomycete, was constructed. The evolutionary history was inferred by using the Maximum Likelihood method based on [2] and an analysis, involving 24 nucleotide sequences with a total of 1072 positions in the final dataset, was conducted in MEGA7 [3].

**Additional File S9.** Phylogenetic tree depicting the relationship of *M. cinnamomea* to other species, including those that were used for comparison of CAZymes in this study. Bootstrap values of > 50%, obtained from 1000 replications, are shown. GenBank accession numbers of 18S rRNA gene sequences are given in parentheses.

**ITS sequences of species from the phylogenetic tree:**

**>Malbranchea cinnamomea FCH 10.5**

TACCTGGTTGATTCTGCCAGTAGTCATATGCTTGTCTCAAAGATTAAGCCATGCATGTCT

AAGTATAAGCAATCTATACGGTGAAACTGCGAATGGCTCATTAAATCAGTTATCGTTTAT

TTGATAGTACCTTACTACATGGATACCCGTGGTAATTCTAGAGCTAATACATGCTAAAAA

CCTCGACTTCGGAAGGGGTGTATTTATTAGATAAAAAACCAATGCCCTTCGGGGCTTGTT

GGTGATTCATAATAACTTCTCGAATCGCATGGCCTTGCGCCGGCGATGGTTCATTCAAAT

TTCTGCCCTATCAACTTTCGATGGTAGGATAGTGGCCTACCATGGTGGCAACGGGTAACG

GGGAATTAGGGTTCGATTCCGGAGAGGGAGCCTGAGAAACGGCTACCACATCCAAGGAAG

GCAGCAGGCGCGCAAATTACCCAATCCCGATACGGGGAGGTAGTGACAATAAATACTGAT

GCAGGGCTCTTTCGGGTCTTGCAATCGGAATGAGTACAATCTAAATCCCTTAACGAGGAA

CAATTGGAGGGCAAGTCTGGTGCCAGCAGCCGCGGTAATTCCAGCTCCAATAGCGTATAT

TAAAGTTGTTGCAGTTAAAAAGCTCGTAGTTGAACCTTGGGCCTGGCTGGCCGGTCCGCC

TCACGGCGTGCACTGGTCCGGCTGGGTCTTTCCTTCTGGGGAGCCCCATGGCCTTCACTG

GCTGTGGGGGGAACCAGGACTTTTACTGTGAAAAAATTAGAGTGTTCAAAGCAGGCCTTT

GCTCGGATACATTAGCATGGAATAATAGAATAGGACGTGCGGTTCTATTTTGTTGGTTTC

TAGGACCGCCGTAATGATTAATAGGGATAGTCGGGGGCGTCAGTATTCGGCTGTCAGAGG

TGAAATTCTTGGATTTGCCGAAGACTAACTACTGCGAAAGCATTCGCCAAGGATGTTTTC

ATTAATCAGGGAACGAAAGTTAGGGGATCGAAGACGATCAGATACCGTCGTAGTCTTAAC

CATAAACTATGCCGACTAGGGATCGGACGGTGTTTCTATGATGACCCGTTCGGCACCTTA

CGAGAAATCAAAGTGTTTGGGTTCTGGGGGGAGTATGGTCGCAAGGCTGAAACTTAAAGA

AATTGACGGAAGGGCACCACCAGGCGTGGAGCCTGCGGCTTAATTTGACTCAACACGGGG

AAACTCACCAGGTCCAGACAAAATAAGGATTGACAGATTGAGAGCTCTTTCTTGATCTTT

TGGATGGTGGTGCATGGCCGTTCTTAGTTGGTGGAGTGATTTGTCTGCTTAATTGCGATA

ACGAACGAGACCTTAACCTGCTAAATAGCCCGACCCACGTTTGTGGGCCGCTGGCTTCTT

AGAGGGACTATCGGCTCAAGCCGATGGAAGTTTGAGGCAATAACAGGTCTGTGATGCCCT

TAGATGTTCTGGGCCGCACGCGCGCTACACTGACAGGGCCAGCGAGTTTATTTCCTTGGC

CGAGAGGTCTGGGTAATCTTGTTAAACCCTGTCGTGCTGGGGATAGAGCATTGCAATTAT

TGCTCTTCAACGAGGAATGCCTAGTAGGCACGAGTCATCAGCTCGTGCCGATTACGTCCC

TGCCCTTTGTACACACCGCCCGTCGCTACTACCGATTGAATGGCTCAGTGAGGCCTCCGG

ACTGGCCCAGGGAGGTTGGCAACGATCGCCCAGGGCCGGAAAGCTGGTCAAACTTGGTCA

TTTAGAGGAAGTAAAAGTCGTAACAAGGTTTCCGTAGGTGAACCTGCGGAAGGATCATTA

**>Yarrowia lipolytica JCM 2320T (AB018158)**

AGTCATATGCTTGTCTCAAAGACTAAGCCATGCATGTCTAAGTATAAACAATTATACAGT

GAAACTGCGAACGGCTCATTAAATCAGTTATCGTTTATTTGATAGTTTTCTACATGGATA

ACCGTGATAACTTCAGAACTAATACATGACAGCCTTCTGGCGTATATATTAGATACAAAC

CAACAGTATGGTGATTCATAATATCTTGTCGAACCGATCTTCGGTGTATCATTCAAATTT

CTGCCCTATCAACTGTCGATGGTAGGATCGTGGCCTACCATGGTAACAACGGGTAACGGG

GAATCAGGGTTCTATTCCGGAGAGGGAGCCTGAGAAACGGCTACCACATCCAAGGAAGGC

AGCAGGCGCGCAAATTACCCAATCCTGACACAGGGAGGTAGTGACAATATATAACGATCC

GGGGCTCTTTGAGTTTCGGAATTGGAATGAGTACAATTTAAACACCTTAACGAGGAACAA

TTGGAGGGCAAGTCTGGTGCCAGCAGCCGCGGTAATTCCAGCTCCAATAGCGTATATTAA

TGTTGTTGCAGTTAAAAAGCTCGTAGTTGAAATTGGGCGGGCTATTAGTTTAGGCCGCTT

CAGGAAGAACTTCTTCCAGTTACTTTGAAAAAATTAGAGTGTTCAACGCAGGTTTCGCCT

GAATATATTAGCATGGAATAACATAACACGACGAGGGTCCATTTTGTTGGCTTGCAAACC

CACGTAATGATTAATAGGGACAGTCGGGGGCGTCAGTATTGTGTTGTCAGAGGTGAAATT

CTTGGATTTACACAAGACTAACTACTGCGAAGCATTCGCCAAGGATGTATTCATTAATCA

AGAACGAAAGTTAGGGGATCAAAGATGATCAGATACCGTCGTAGTCTTAACCGTAAACTA

TGCCGACTGAGAATGGGTACCGCTTATACGGTATCCGCGCTCTACGAGAAATCAAAGTGA

TCAGGTTCTGGGGGGAGTATGGTCGCAAGGCTGAAACTTAAAGGAATTGACGGAAGGGCA

CCACCAGGAGTGGGACTGCGGCTTAATTTGACTCAACACGGGGAAACTCACCAGGTCCAG

ACACAATAAGGATTGACAGATTGATAGCTCTTTCTTGATTTTGTGGGTGGTGGTGCATGG

CCGTTCTTAGTTGGTGGAGTGATTTGTCTGCTTAATTGCGATAACGAACGAGACCTTGAC

CTACTAAATAGCTCTACNNCGATTGCAGGTAGCTAGCTTCTTAGAGGGACTATCTATTAC

AAGTAGATGGAAGTTCGAGGCAATAACAGGTCTGTGATGCCCTTAGACGTTCTGGGCCGC

ACGCGCGCTACACTGACGGAGCCAGCGAGTCGACCAAGCCCGAGAGGGCTAGGTAATCTT

GTGAAACTCCGTCGTGCTGGGGATAGAGCATTGCAATTATTGCTCTTCAACGAGGAATTC

CTAGTAAGCGCAAGTCATCAGCTTGCGTTGATTACGTCCCTGCCCTTTGTACACACCGCC

CGTCGCTACTACCGATTGAATGGTTTAGTGAGACCTTGGGAGGGCGAGATGAGGGGGGCA

ACCCCTTTTGAACATCCAAACTTGGTCAAACTTGATTATTTAGAGGAAGTAAAAGTCGTA

ACAAGGTTTCCGTAGGTGAACCTGCGG

**>Saccharomyces cerevisiae NRRL Y-12632T (EU011664)**

ATGTCTAAGTATAAGCAATTTATACAGTGAAACTGCGAATGGCTCATTAAATCAGTTATC

GTTTATTTGATAGTTCCTTTACTACATGGTATAACTGTGGTAATTCTAGAGCTAATACAT

GCTTAAAATCTCGACCCTTTGGAAGAGATGTATTTATTAGATAAAAAATCAATGTCTTCG

GACTCTTTGATGATTCATAATAACTTTTCGAATCGCATGGCCTTGTGCTGGCGATGGTTC

ATTCAAATTTCTGCCCTATCAACTTTCGATGGTAGGATAGTGGCCTACCATGGTTTCAAC

GGGTAACGGGGAATAAGGGTTCGATTCCGGAGAGGGAGCCTGAGAAACGGCTACCACATC

CAAGGAAGGCAGCAGGCGCGCAAATTACCCAATCCTAATTCAGGGAGGTAGTGACAATAA

ATAACGATACAGGGCCCATTCGGGTCTTGTAATTGGAATGAGTACAATGTAAATACCTTA

ACGAGGAACAATTGGAGGGCAAGTCTGGTGCCAGCAGCCGCGGTAATTCCAGCTCCAATA

GCGTATATTAAAGTTGTTGCAGTTAAAAAGCTCGTAGTTGAACTTTGGGCCCGGTTGGCC

GGTCCGATTTTTTCGTGTACTGGATTTCCAACGGGGCCTTTCCTTCTGGCTAACCTTGAG

TCCTTGTGGCTCTTGGCGAACCAGGACTTTTACTTTGAAAAAATTAGAGTGTTCAAAGCA

GGCGTATTGCTCGAATATATTAGCATGGAATAATAGAATAGGACGTTTGGTTCTATTTTG

TTGGTTTCTAGGACCATCGTAATGATTAATAGGGACGGTCGGGGGCATCAGTATTCAATT

GTCAGAGGTGAAATTCTTGGATTTATTGAAGACTAACTACTGCGAAAGCATTTGCCAAGG

ACGTTTTCATTAATCAAGAACGAAAGTTAGGGGATCGAAGATGATCAGATACCGTCGTAG

TCTTAACCATAAACTATGCCGACTAGGGATCGGGTGGTGTTTTTTTAATGACCCACTCGG

CACCTTACGAGAAATCAAAGTCTTTGGGTTCTGGGGGGAGTATGGTCGCAAGGCTGAAAC

TTAAAGGAATTGACGGAAGGGCACCACCAGGAGTGGAGCCTGCGGCTTAATTTGACTCAA

CACGGGGAAACTCACCAGGTCCAGACACAATAAGGATTGACAGATTGAGAGCTCTTTCTT

GATTTTGTGGGTGGTGGTGCATGGCCGTTCTTAGTTGGTGGAGTGATTTGTCTGCTTAAT

TGCGATAACGAACGAGACCTTAACCTACTAAATAGTGGTGCTAGCATTTGCTGGTTATCC

ACTTCTTAGAGGGACTATCGGTTTCAAGCCGATGGAAGTTTGAGGCAATAACAGGTCTGT

GATGCCCTTAGACGTTCTGGGCCGCACGCGCGCTACACTGACGGAGCCAGCGAGTCTAAC

CTTGGCCGAGAGGTCTTGGTAATCTTGTGAAACTCCGTCGTGCTGGGGATAGAGCATTGT

AATTATTGCTCTTCAACGAGGAATTCCTAGTAAGCGCAAGTCATCAGCTTGCGTTGATTA

CGTCCCTGCCCTTTGTACACACCGCCCGTCGCTAGTACCGATTGAATGGCTTAGTGAGGC

CTCAGGATCTGCTTAGAGAAGGGGGCAACTCCATCTCAGAGCGGAGAATTTGGACAAACT

TGGTCATTTAGAGGAACTAAAAGTCGTAACAAGGTTTCCGTAGGTGAACC

**>Kluyveromyces lactis NRRL Y-8279 (AY046264)**

AGTCATATGCTTGTCTCAAAGATTAAGCCATGCATGTCTAAGTATAAGCAATTTATACAG

TGAAACTGCGAATGGCTCATTAAATCAGTTATCGTTTATTTGATAGTTCCTTTACTACAT

GGATATCTGTGGTAATTCTAGAGCTAATACATGCTTAAAATCTCGACCCTTTGGAAGAGA

TGTATTTATTAGATAAAAAATCAATGTCTTCGGACTCCTTGATGATTCATAATAACTTTT

CGAATCGCATGGCCTTGTGCTGGCGATGGTTCATTCAAATTTCTGCCCTATCAACTTTCG

ATGGTAGGATAGTGGCCTACCATGGTTTCAACGGGTAACGGGGAATAAGGGTTCGATTCC

GGAGAGGGAGCCTGAGAAACGGCTACCACATCCAAGGAAGGCAGCAGGCGCGCAAATTAC

CCAATCCTAATTCAGGGAGGTAGTGACAATAAATAACGATACAGGGCCCATTCGGGTCTT

GTAATTGGAATGAGTACAATGTAAATACCTTAACGAGGAACAACTGGAGGGCAAGTCTGG

TGCCAGCAGCCGCGGTAATTCCAGCTCCAGTAGCGTATATTAAAGTTGTTGCAGTTAAAA

AGCTCGTAGTTGAACTTTGGGTCTGGTTGTCCGGTCCGACTTTATGTCGCGCACTGGTTT

TCAACCGGATCTTTCCTTCTGGCTAACCTGTACTCCTTGTGGGTGCAGGCGAACCAGGAC

TTTTACTTTGAAAAAATTAGAGTGTTCAAAGCAGGCGAAAGCTCGAATATATTAGCATGG

AATAATGGAATAGGACGTTTGGTTCTATTTTGTTGGTTTCTAGGACCATCGTAATGATTA

ATAGGGACGGTCGGGGGCATCAGTATTCAATTGTCAGAGGTGAAATTCTTGGATTTATTG

AAGACTAACTACTGCGAAAGCATTTGCCAAGGACGTTTTCATTAATCAAGAACGAAAGTT

AGGGGATCGAAGATGATCAGATACCGTCGTAGTCTTAACCATAAACTATGCCGACTAGGG

ATCGGGTGGTGTTTTTCTTATGACCCACTCGGCACCTTACGAGAAATCAAAGTCTTTGGG

TTCTGGGGGGAGTATGGTCGCAAGGCTGAAACTTAAAGGAATTGACGGAAGGGCACCACC

AGGAGTGGAGCCTGCGGCTTAATTTGACTCAACACGGGGAAACTCACCAGGTCCAGACAC

AATAAGGATTGACAGATTGAGAGCTCTTTCTTGATTTTGTGGGTGGTGGTGCATGGCCGT

TCTTAGTTGGTGGAGTGATTTGTCTGCTTAATTGCGATAACGAACGAGACCTTAACCTAC

TAAATAGGGTTGCTGGCACTTGCCGGTTGACTCTTCTTAGAGGGACTATCGGTTTCAAGC

CGATGGAAGTTTGAGGCAATAACAGGTCTGTGATGCCCTTAGACGTTCTGGGCCGCACGC

GCGCTACACTGACGGAGCCAGCGAGTACAACCTTGGCCGAGAGGTCTGGGTAATCTTGTG

AAACTCCGTCGTGCTGGGGATAGAGCATTGTAATTATTGCTCTTCAACGAGGAATTCCTA

GTAAGCGCAAGTCATCAGCTTGCGTTGATTACGTCCCTGCCCTTTGTACACACCGCCCGT

CGCTAGTACCGATTGAATGGCTTAGTGAGGCCTCAGGATTTGCTTAGAGAAGGGGGCAAC

TCCATCTCAGAGCGAAGAATCTGGTCAAACTTGGTCATTTAGAGGAACTAAAAGTCGTAA

CAAGGTTT

**>Arthrobotrys oligospora ATCC 24927 (AJ001986)**

AGCCATGCATGTCTAAGTATAAGCAACTATACAGTGAAACTGCGAATGGCTCATTAAATC

AGTTATCGTTTATTTGATAGTACCTTACTACTTGGATAACCGTGGTAATTCTAGAGCTAA

TACATGCTAAAAATCCCGACCTCCGGAAGGGATGTATTTATTAGATAAAAAACCAATGCC

TTCGGGCTCCTTGGTGATTCATGATAACTTAACGAATCGCATGGCCTTGCGCCGGCGATG

GTTCATTCAAATTTCTGCCCTATCAACTTTCGATGGTAGGATAGTGGCCTACCATGGTTT

CAACGGGTAACGGGGAAATTAGGGTTCGATTCCGGAGAGGGAGCCTGAGTAAACGGCTAC

CACATCCAAGGAAGGCAGCAGGCGCGCAAATTACCCAATCCCGATTCGGGGNAGGTAGTG

ACAATAAATACTGATACAGGGCTCTTTTGGGTCTTGTAATTGGAATGAGTACAATTTAAA

TCCCTTAACGAGGAACAATTGGAGGGCAAGTCTGGTGCCAGCAGCCGCGGTAATTCCAGC

TCCAATAGCGTATATTAAAGTTGTTGCAGTTAAAAAGCTCGTAGTTGAACCTTGGGTTTG

GCTGCTCGGTCCGCCTAACCGCGTGCACTGATGCGGCCGGATCTTTCCTTCTGGCTAACC

TCATGCCCTTTACTGGGTGTGCTGGGGATCCAGGACTTTTACTTTGAAAAAATTAGAGTG

TTCAAAGCAGGCCTTTGCTCGAATACATTAGCATGGAATAATAGAATAGGACGGCGGTTC

TATTTTGTTGGTTTCTAGAGCCACCGTAATGATTAATAGGGATAGTCGGGGGCATCAGTA

TTCAATTGTCAGAGGTGAAATTCTTGGATTTATTGAAGACTAACTACTGCGAAAGCATTT

GCCAAGGATGTTTTCATTAATCAGTGAACGAAAGTTAGGGGATCGAAGACGATCAGATAC

CGTCGTAGTCTTAACCATAAACTATGCCGACTAGGGATCGGGCGGTGTTCAACTTATGAC

CCGCTCGGCACCTTACGAGAAATCAAAGTTTTTGGGTTCTGGGGGGAGTATGGTCNCAAG

GCTGAAACTTAAAGGAATTGACGGAAGGGCACCACCAGATGTGGAGCCTGCGGCTTAATT

TGACTCAACACGGGGAAACTCACCAGGTCCAGACACATTAAGGATTGACAGATTGAGAGC

TCTTTCTTGATTATGTGGGTGGTGGTGCATGGCCGTTCTTAGTTGGTGGAGTGATTTGTC

TGCTTAATTGCGATAACGAACGAGACCTTAACCTGCTAAATAGCCTGGCTAGCTTTTGCT

GGTCACCGGCTTCTTAGAGGGACTATCGGCTCAAGCCGATGGAAGTTTGAGGCAATAACA

GGTCTGTGATGCCCTTAGATGTTCTGGGCCGCACGCGCGCTACACTGACAGAGCCAACGA

GTATAACCCTTAGCCGAGAGGTTTAGGTAATCTTGTTAAACTCTGTCGTGCTGGGGATAG

GGCATTGCAATTATTGCCCTTCAACGAGGAATATCTAGTAAGCGCAAGTCATCAGCTTGC

GTTGATTACGTCCCTGCCCTTTGTACACACCGCCCGTCGCTACTACCGATTGAATGGCTC

AGTGAGGCCTTCGGACTGGCTCCAGGAGGTTGGCAACAACCACCCAGAGCCGGAAA

**>Penicillium chrysogenum CBS 306.48T (GU733359)**

GCTCATTAAATCAGTTATCGTTTATTTGATAGTACCTTACTACATGGATACCTGTGGTAA

ATTCTAGAGCTAATACATGCTAAAAACCCCCGACTTCAGGAAGGGGGTGTATTTATTAGA

TAAAAAACCAACGCCCTTTCGGGGCTCCTTGGTGAATCATAATAACTTAACGAATCGCAT

GGCCTTGCGCCGGCGATGGTTCATTCAAATTTCTGCCCTATCAACTTTCGATGGTAGGAT

AGTGGCCTACCATGGTGGCAACGGGTAACGGGGAATTAGGGTTCGATTCCGGAGAGGGAG

CCTGAGAAACGGCTACCACATCCAAGGAAGGCAGCAGGCGCGCAAATTACCCAATCCCGA

TACGGGGAGGTAGTGACAATAAATACTGATACGGGGCTCTTTTGGGTCTCGTAATTGGAA

TGAGAACAATTTAAATCCCTTAACGAGGAACAATTGGAGGGCAAGTCTGGTGCCAGCAGC

CGCGGTAATTCCAGCTCCAATAGCGTATATTAAAGTTGTTGCAGTTAAAAAGCTCGTAGT

TGAACCTTGGGTCTGGCTGGCCGGTCCGCCTCACCGCGAGTACTGGTCCGGCTGGACCTT

TCCTTCTGGGGAACCTCATGGCCTTCACTGGCTGTGGGGGGAACCAGGACTTTTACTGTG

AAAAAATTAGAGTGTTCAAAGCAGGCCTTTGCTCGAATACATTAGCATGGAATAATAGAA

TAGGACGTGTGGTTCTATTTTGTTGGTTTCTAGGACCGCCGTAATGATTAATAGGGATAG

TCGGGGGCGTCAGTATTCAGCTGTCAGAGGTGAAATTCTTGGATTTGCTGAAGACTAACT

ACTGCGAAAGCATTCGCCAAGGATGTTTTCATTAATCAGGGAACGAAAGTTAGGGGATCG

AAGACGATCAGATACCGTCGTAGTCTTAACCATAAACTATGCCGACTAGGGATCGGACGG

GATTCTATAATGACCCGTTCGGCACCTTACGAGAAATCAAAGTTTTTGGGTTCTGGGGGG

AGTATGGTCGCAAGGCTGAAACTTAAAGAAATTGACGGAAGGGCACCACAAGGCGTGGAG

CCTGCGGCTTAATTTGACTCAACACGGGGAAACTCACCAGGTCCAGACAAAATAAAGGAT

TGACAGATTGAGAGCTCTTTCTTGATCTTTTGGATGGTGGTGCATGGCCGTTCTTAGTTG

GTGGAGTGATTTGTCTGCTTAATTGCGATAAACGAACGAGACCTCGGCCCTTAAATAGCC

CGGTCCGCATTTGCGGGCCGCTGGCTTCTTAGGGGGACTATCGGCTCAAGCCGATGGAAG

TGCGCGGCAATAACAGGGTCTGTGATGCCCTTAGATGTTCTGGGCCCGCACGCGCGCTAC

ACTGACAGGGCCAGCGAGTACATCACCTTAAACCGAGAGGTTTGGGTAATCTTGTTAAAC

CCTGTCGTGCTGGGGATAGAGCATTGCAATTATTGCTTTTCAACGAGGAATGCCTAGTAG

GCACGAGTCATCAGCTCGTGCCGATTACGTCCCTGCCCTTTGTACACACCGCCCGTCGCT

ACTACCGATTGAATGGCTCAGTGAGGCCTTGGGATTGG

**>Aspergillus nidulans ATCC 10074 (AB008403)**

AAAGATTAAGCCATGCATGTCTAAGTATAAGCAATCTATACTGTGAAACTGCGAATGGCT

CATTAAATCAGTTATCGTTTATTTGATAGTACCTTACTACATGGATACCTGTGGTAATTC

TAGAGCTAATACATGCTAAAAACCCCGACTTCGGGAGGGGTGTATTTATTAGATAAAAAA

CCAATGCCCCTCGGGGCTCCTTGGTGATTCATAATAACTTAACGAATCGCATGGCCTTGC

GCCGGCGATGGTTCATTCAAATTTCTGCCCTATCAACTTTCGATGGTAGGATAGTGGCCT

ACCATGGTGGCAACGGGTAACGGGGAATTAGGGTTCGATTCCGGAGAGGGAGCCTGAGAA

ACGGCTACCACATCCAAGGAAGGCAGCAGGCGCGCAAATTACCCAATCCCGACACGGGGA

GGTAGTGACAATAAATACTGATACGGGGCTCTTTTGGGTCTCGTAATTGGAATGAGAACA

ATTTAAATCCCTTAACGAGGAACAATTGGAGGGCAAGTCTGGTGCCAGCAGCCGCGGTAA

TTCCAGCTCCAATAGCGTATATTAAAGTTGTTGCAGTTAAAAAGCTCGTAGTTGAACCTT

GGGTCTGGCTGGCCGGTCCGCCTCACCGCGAGTACTGGTCCGGCTGGACCTTTCCTTCTG

GGGAACCCCATGGCCTTCACTGGCTGTGGGGGGAACCAGGACTTTTACTGTGAAAAAATT

AGAGTGTTCAAAGCAGGCCTTTGCTCGGATACATTAGCATGGAATAATAGAATAGGACGT

GCGGTTCTATTTTGTTGGTTTCTAGGACCGCCGTAATGATTAATAGGGATAGTCGGGGGC

GTCAGTATTCAGCTGTCAGAGGTGAAATTCTTGGATTTGCTGAAGACTAACTACTGCGAA

AGCATTCGCCAAGGATGTTTTCATTAATCAGGGAACGAAAGTTAGGGGATCGAAGACGAT

CAGATACCGTCGTAGTCTTAACCATAAACTATGCCGACTAGGGATCGGGCGGCGTTTCTT

TTATGACCCGCTCGGCACCTTACGAGAAATCAAAGTTTTTGGGTTCTGGGGGGAGTATGG

TCGCAAGGCTGAAACTTAAAGAAATTGACGGAAGGGCACCACAAGGCGTGGAGCCTGCGG

CTTAATTTGACTCAACACGGGGAAACTCACCAGGTCCAGACAAAATAAGGATTGACAGAT

TGAGAGCTCTTTCTTGATCTTTTGGATGGTGGTGCATGGCCGTTCTTAGTTGGTGGAGTG

ATTTGTCTGCTTAATTGCGATAACGAACGAGACCTCGGCCCTTAAATAGCCCGGTCCGCG

TCCGCGGGCCGCTGGCTTCTTAGGGGGACTATCGGCTCAAGCCGATGGAAGTGCGCGGCA

ATAACAGGTCTGTGATGCCCTTAGATGTTCTGGGCCGCACGCGCGCTACACTGACAGGGC

CAGCGAGTACATCACCTTGGCCGAGAGGCCCGGGTAATCTTGTTAAACCCTGTCGTGCTG

GGGATAGAGCATTGCAATTATTGCTCTTCAACGAGGAATGCCTAGTAGGCACGAGTCATC

AGCTCGTGCCGATTACGTCCCTGCCCTTTGTACACACCGCCCGTCGCTACTACCGATTGA

ATGGCTCGGTGAGGCCTCCGGACTGGCTCAGGAGGGTTGGCAACGACCCCCCCGAGCCGG

AAAGCTGGTCAAACCCGGTCATTTAGAGGAAGTAAAAGTCGTAACAAGGTTTC

**>Aspergillus niger CBS 513.88 (An03c0110)**

CTGGTTGATTCTGCCAGTAGTCATATGCTTGTCTCAAAGATTAAGCCATGCATGTCTAAG

TATAAGCACTTTATACTGTGAAACTGCGAATGGCTCATTAAATCAGTTATCGTTTATTTG

ATAGTACCTTACTACATGGATACCTGTGGTAATTCTAGAGCTAATACATGCTGAAAACCT

CGACTTCGGAAGGGGTGTATTTATTAGATAAAAAACCAATGCCCTTCGGGGCTCCTTGGT

GAATCATAATAACTTAACGAATCGCATGGCCTTGCGCCGGCGATGGTTCATTCAAATTTC

TGCCCTATCAACTTTCGATGGTAGGATAGTGGCCTACCATGGTGGCAACGGGTAACGGGG

AATTAGGGTTCGATTCCGGAGAGGGAGCCTGAGAAACGGCTACCACATCCAAGGAAGGCA

GCAGGCGCGCAAATTACCCAATCCCGACACGGGGAGGTAGTGACAATAAATACTGATACG

GGGCTCTTTTGGGTCTCGTAATTGGAATGAGTACAATCTAAATCCCTTAACGAGGAACAA

TTGGAGGGCAAGTCTGGTGCCAGCAGCCGCGGTAATTCCAGCTCCAATAGCGTATATTAA

AGTTGTTGCAGTTAAAAAGCTCGTAGTTGAACCTTGGGTCTGGCTGGCCGGTCCGCCTCA

CCGCGAGTACTGGTCCGGCTGGACCTTTCCTTCTGGGGAATCTCATGGCCTTCACTGGCT

GTGGGGGGAACCAGGACTTTTACTGTGAAAAAATTAGAGTGTTCAAAGCAGGCCTTTGCT

CGAATACATTAGCATGGAATAATAGAATAGGACGTGCGGTTCTATTTTGTTGGTTTCTAG

GACCGCCGTAATGATTAATAGGGATAGTCGGGGGCGTCAGTATTCAGCTGTCAGAGGTGA

AATTCTTGGATTTGCTGAAGACTAACTACTGCGAAAGCATTCGCCAAGGATGTTTTCATT

AATCAGGGAACGAAAGTTAGGGGATCGAAGACGATCAGATACCGTCGTAGTCTTAACCAT

AAACTATGCCGACTAGGGATCGGACGGTGTTTCTATTATGACCCGTTCGGCACCTTACGA

GAAATCAAAGTTTTTGGGTTCTGGGGGGAGTATGGTCGCAAGGCTGAAACTTAAAGAAAT

TGACGGAAGGGCACCACCAGGCGTGGAGCCTGCGGCTTAATTTGACTCAACACGGGGAAA

CTCACCAGGTCCAGACAAAATAAGGATTGACAGATTGAGAGCTCTTTCTTGATCTTTTGG

ATGGTGGTGCATGGCCGTTCTTAGTTGGTGGAGTGATTTGTCTGCTTAATTGCGATAACG

AACGAGACCTCGGCCCTTAAATAGCCCGGTCCGCATTTGCGGGCCGCTGGCTTCTTAGGG

GGACTATCGGCTCAAGCCGATGGAAGTGCGCGGCAATAACAGGTCTGTGATGCCCTTAGA

TGTTCTGGGCCGCACGCGCGCTACACTGACAGGGCCAGCGAGTACATCACCTTGGCCGAG

AGGTCTGGGTAATCTTGTTAAACCCTGTCGTGCTGGGGATAGAGCATTGCAATTATTGCT

CTTCAACGAGGAATGCCTAGTAGGCACGAGTCATCAGCTCGTGCCGATTACGTCCCTGCC

CTTTGTACACACCGCCCGTCGCTACTACCGATTGAATGGCTCGGTGAGGCCTTCGGACTG

GCTCAGGAGGGTTGGCAACGACCCCCCAGAGCCGGAAAGTTGGTCAAACCCGGTCATTTA

GAGGAAGTAAAAGTCGTAACAAGGTTTCCGTAGGTGAACCTGCGGAAGGATCATTACCGA

GT

**>Aspergillus oryzae ATCC 1011T (D63698)**

AAAGATTAAGCCATGCATGTCTAAGTATAAGCACTTTATACTGTGAAACTGCGAATGGCT

CATTAAATCAGTTATCGTTTATTTGATAGTACCTTACTACATGGATACCTGTGGTAATTC

TAGAGCTAATACATGCTAAAAACCTCGACTTCGGAAGGGGTGTATTTATTAGATAAAAAA

CCAATGCCCTTCGGGGCTCCTTGGTGATTCATAATAACTTAACGAATCGCATGGCCTTGC

GCCGGCGATGGTTCATTCAAATTTCTGCCCTATCAACTTTCGATGGTAGGATAGTGGCCT

ACCATGGTGGCAACGGGTAACGGGGAATTAGGGTTCGATTCCGGAGAGGGAGCCTGAGAA

ACGGCTACCACATCCAAGGAAGGCAGCAGGCGCGCAAATTACCCAATCCCGACACGGGGA

GGTAGTGACAATAAATACTGATACGGGGCTCTTTTGGGTCTCGTAATTGGAATGAGTACA

ATCTAAATCCCTTAACGAGGAACAATTGGAGGGCAAGTCTGGTGCCAGCAGCCGCGGTAA

TTCCAGCTCCAATAGCGTATATTAAAGTTGTTGCAGTTAAAAAGCTCGTAGTTGAACCTT

GGGTCTGGCTGGCCGGTCCGCCTCACCGCGAGTACTGGTCCGGCTGGACCTTTCCTTCTG

GGGAACCTCATGGCCTTCACTGGCTGTGGGGGGAACCAGGACTTTTACTGTGAAAAAATT

AGAGTGTTCAAAGCAGGCCTTTGCTCGAATACATTAGCATGGAATAATAGAATAGGACGT

GCGGTTCTATTTTGTTGGTTTCTAGGACCGCCGTAATGATTAATAGGGATAGTCGGGGGC

GTCAGTATTCAGCTGTCAGAGGTGAAATTCTTGGATTTGCTGAAGACTAACTACTGCGAA

AGCATTCGCCAAGGATGTTTTCATTAATCAGGGAACGAAAGTTAGGGGATCGAAGACGAT

CAGATACCGTCGTAGTCTTAACCATAAACTATGCCGACTAGGGATCGGGCGGTGTTTCTA

TGATGACCCGCTCGGCACCTTACGAGAAATCAAAGTTTTTGGGTTCTGGGGGGAGTATGG

TCGCAAGGCTGAAACTTAAAGAAATTGACGGAAGGGCACCACAAGGCGTGGAGCCTGCGG

CTTAATTTGACTCAACACGGGGAAACTCACCAGGTCCAGACAAAATAAGGATTGACAGAT

TGAGAGCTCTTTCTTGATCTTTTGGATGGTGGTGCATGGCCGTTCTTAGTTGGTGGAGTG

ATTTGTCTGCTTAATTGCGATAACGAACGAGACCTCGGCCCTTAAATAGCCCGGTCCGCG

TTTGCGGGCCGCTGGCTTCTTAGGGGGACTATCGGCTCAAGCCGATGGAAGTGCGCGGCA

ATAACAGGTCTGTGATGCCCTTAGATGTTCTGGGCCGCACGCGCGCTACACTGACAGGGC

CAGCGAGTACATCACCTTGGCCGAGAGGTCCGGGTAATCTTGTTAAACCCTGTCGTGCTG

GGGATAGAGCATTGCAATTATTGCTCTTCAACGAGGAATGCCTAGTAGGCACGAGTCATC

AGCTCGTGCCGATTACGTCCCTGCCCTTTGTACACACCGCCCGTCGCTACTACCGATTGA

ATGGCTCGGTGAGGCCTTCGGACTGGCCCAGGAGGGTTGGCAACGACCCCCCAGGGCCGG

AAAGTTGGTCAAACCCGGTCATTTAGAGGAAGTAAAAGTCGTAACAAGGTTTCCGTAGGT

GAACCTGCGGAAGGATCATTA

**>Leptosphaeria maculans Leroy (U04233)**

TACCTGGTTGATTCTGCCAGTAGTCATATGCTTGTCTCAAAGATTAAGCCATGCATGTCT

AAGTATAAGCAATTATACCGTGAAACTGCGAATGGCTCATTAAATCAGTTATCGTTTATT

TGATAGTACCTTACTACTTGGATAACCGTGGTAATTCTAGAGCTAATACATGCTAAAAAC

CCCGACTTCGGAAGGGGTGTATTTATTAGATAAAAAACCAACGCCCTTCGGGGCTTCTTG

GTGATTCATGATAACTTTACGGATCGCATGGCCTTGCGCCGGCGACGGTTCATTCAAATT

TCTGCCCTATCAACTTTCGATGGTAAGGTATTGGCTTACCATGGTTTCAACGGGTAACGG

GGAATTAGGGTTCGATTCCGGAGAGGGAGCCTGAGAAACGGCTACCACATCCAAGGAAGG

CAGCAGGCGCGCAAATTACCCAATCCCGACACGGGGAGGTAGTGACAATAAATACTGATA

CAGGGCTCTTTTGGGTCTTGTAATTGGAATGAGTACAATTTAAACCTCTTAACGAGGAAC

AATTGGAGGGCAAGTCTGGTGCCAGCAGCCGCGGTAATTCCAGCTCCAATAGCGTATATT

AAAGTTGTTGCAGTTAAAAAGCTCGTAGTTGAAACTTGGGTCTGGCTGGCAGGTCCGCCT

CACCGCGTGTACTTGTCCGGCCGGGCCTTCCTTCTGGAGAACCTCATGCCCTTCACTGGG

CGTGTTGGGGACCAGGACTTTTACTTTGAAAAAATTAGAGTGTTCAAAGCAGGCCTTTGC

TCGAATACGTTAGCATGGAATAATAGAATAGGACGTGCGGTCCTATTTTGTTGGTTTCTA

GGACCGCCGTAATGATTAATAGGGACAGTCGGGGGCATCAGTATTCAATTGTCAGAGGTG

AAATTCTTGGATTTATTGAAGACTAACTACTGCGAAAGCATTTGCCAAGGATGTTTTCAT

TAATCAGTGAACGAAAGTTAGGGGATCGAAGACGATCAGATACCGTCGTAGTCTTAACCG

TAAACTATGCCGACTAGGGATCGGGCGATGTTCTTTTTCTGACTCGCTCGGCACCTTACG

AGAAATCAAAGTTTTTGGGTTCTGGGGGGAGTATGGTCGCAAGGCTGAAACTTAAAGAAA

TTGACGGAAGGGCACCACCAGGCGTGGAGCCTGCGGCTTAATTTGACTCAACACGGGGAA

ACTCACCAGGTCCAGATGAAATAAGGATTGACAGATTGAGAGCTCTTTCTTGATTTTTCA

GGTGGTGGTGCATGGCCGTTCTTAGTTGGTGGAGTGATTTGTCTGCTTAATTGCGATAAC

GAACGAGACCTTAACCTGCTAAATAGCCAGGCTAGCTTTGGCTGGTCGCCGGCTTCTTAG

AGGGACTATCGGCTCAAGCCGATGGAAGTTTGAGGCAATAACAGGTCTGTGATGCCCTTA

GATGTTCTGGGCCGCACGCGCGCTACACTGACAGAGCCAACGAGTTCTTCACCTTGACCG

AAAGGTCTGGGTAATCTTGTTAAACTCTGTCGTGCTGGGGATAGAGCATTGCAATTATTG

CTCTTCAACGAGGAATGCCTAGTAAGCGCGTGTCATCAGCATGCGTTGATTACGTCCCTG

CCCTTTGTACACACCGCCCGTCGCTACTACCGATTGAATGGCTCAGTGAGGCCTTCGGAC

TGGCTCGGGGAGGTTGCCAACGACCACCCTGAGCCGGAAAGTTCGTCAAACTCGGTCATT

TAGAGGAAGTAAAAGTCGTAACAAGGTTTCCGTAGGTGAACCTGCGGAAGGATCATTA

**>Thielavia terrestris NRRL 8126 (CP003011)**

CCTGGTTGATTCTGCCAGTAGTCATATGCTTGTCTCAAAGATTAAGCCATGCATGTCTAA

GTATAAGCAATTTATACAGCGAAACTGCGAATGGCTCATTAAATCAGTTATCGTTTATTT

GATAGTACCTTACTACATGGATAACCGTGGTAATTCTAGAGCTAATACATGCTGAAAATC

CCGACTTCGGAAGGGATGTATTTATTAGATTAAAAACCAATGCCCCTCGGGGCTCTCTGG

TGATTCATAATAACTTCTCGAATCGCACGGCCTTGCGCCGGCGATGGTTCATTCAAATTT

CTGCCCTATCAACTTTCGACGGCTGGGTCTTGGCCAGCCGTGGTCACAACGGGTAACGGA

GGGTTAGGGCTCGACCCCGGAGAAGGAGCCTGAGAAACGGCTACTACATCCAAGGAAGGC

AGCAGGCGCGCAAATTACCCAATCCCGACACGGGGAGGTAGTGACAATAAATACTGATAC

AGGGCTCTTTCGGGTCTTGTAATTGGAATGAGTACAATTTAAATCCCTTAACGAGGAACA

ATTGGAGGGCAAGTCTGGTGCCAGCAGCCGCGGTAATTCCAGCTCCAATAGCGTATATTA

AAGTTGTTGAGGTTAAAAAGCTCGTAGTTGAACCTTGGGCCTAGCCGGCCGGTCCGCCTC

ACCGCGTGCACTGGCTCGGCTGGGTCTTTCCTTCTGGGGAGCCGCATGCCCTTCACTGGG

CGTGCCGGGGAACCAGGACTTTTACTCTGAACAAATTAGATCGCTTAAAGAAGGCCTATG

CTCGAATACGTTAGCATGGAATAATAGAATAGGACGTGTGGTTCTATTTTGTTGGTTTCT

AGGACCGCCGTAATGATTAATAGGGACAGTCGGGGGCATCAGTATTCAATTGTCAGAGGT

GAAATTCTTGGATTTATTGAAGACTAACTACTGCGAAAGCATTTGCCAAGGATGTTTTCA

TTAATCAGGAACGAAAGTTAGGGGATCGAAGACGATCAGATACCGTCGTAGTCTTAACCA

TAAACTATGCCGATTAGGGATCGGACGGCGTTATTTTTTGACCCGTTCGGCACCTTACGA

TAAATCAAAATGTTTGGGCTCCTGGGGGAGTATGGTCGCAAGGCTGAAACTTAAAGAAAT

TGACGGAAGGGCACCACCAGGGGTGGAGCCTGCGGCTTAATTTGACTCAACACGGGGAAA

CTCACCAGGTCCAGACACGATGAGGATTGACAGATTGAGAGCTCTTTCTTGATTTCGTGG

GTGGTGGTGCATGGCCGTTCTTAGTTGGTGGAGTGATTTGTCTGCTTAATTGCGATAACG

AACGAGACCTTAACCTGCTAAATAGCCCGTATTGCTTTGGCAGTACGCCGGCTTCTTAGA

GGGACTATCGGCTCAAGCCGATGGAAGTTTGAGGCAATAACAGGTCTGTGATGCCCTTAG

ATGTTCTGGGCCGCACGCGCGCTACACTGACAGAGCCAGCGAGTACTCCCTTGGCCGGAA

GGCCCGGGTAATCTTGTTAAACTCTGTCGTGCTGGGGATAGAGCATTGCAATTATTGCTC

TTCAACGAGGAATCCCTAGTAAGCGCAAGTCATCAGCTTGCGTTGATTACGTCCCTGCCC

TTTGTACACACCGCCCGTCGCTACTACCGATTGAATGGCTCAGTGAGGCTTTCGGACTGG

CCCAGAGAGGTCGGCAACGACCACTCAGGGCCGGAAAGTTATCCAAACTCGGTCATTTAG

AGGAAGTAAAAGTCGTAACAAGGTCTCCGTTGGTGAACCAGCGGAGG

**>Myceliophthora thermophila ATCC 42464 (CP003008)**

CCTGGTTGATTCTGCCAGTAGTCATATGCTTGTCTCAAAGATTAAGCCATGCATGTCTAA

GTATAAGCAATTATACAGCGAAACTGCGAATGGCTCATTAAATCAGTTATCGTTTATTTG

ATAGTACCTTACTACATGGATAACCGTGGTAATTCTAGAGCTAATACATGCTAAAAATCC

CGACTTCGGAAGGGATGTATTTATTAGATTAAAAACCAATGCCCTCCGGGGCTCTCTGGT

GATTCATGATAACTTCTCGAATCGCACGGCCTTGCGCCGGCGATGGTTCATTCAAATTTC

TGCCCTATCAACTTTCGACGGCTGGGTCTTGGCCAGCCGTGGTGACAACGGGTAACGGAG

GGTTAGGGCTCGACCCCGGAGAAGGAGCCTGAGAAACGGCTACTACATCCAAGGAAGGCA

GCAGGCGCGCAAATTACCCAATCCCGACACGGGGAGGTAGTGACAATAAATACTGATACA

GGGCTCTTTTGGGTCTTGTAATTGGAATGAGTACAATTTAAATCCCTTAACGAGGAACAA

TTGGAGGGCAAGTCTGGTGCCAGCAGCCGCGGTAATTCCAGCTCCAATAGCGTATATTAA

AGTTGTTGAGGTTAAAAAGCTCGTAGTTGAACCTTGGGCCTAGCCGGCCGGTCCGCCTCA

CCGCGTGCACTGGCTCGGCTGGGTCTTTCCTTCTGGAGAACCGCATGCCCTTCACTGGGT

GTGCCGGGGAACCAGGACTTTTACTCTGAACAAATTAGATCGCTTAAAGAAGGCCTATGC

TCGAATACATTAGCATGGAATAATAGAATAGGACGTGTGGTTCTATTTTGTTGGTTTCTA

GGACCGCCGTAATGATTAATAGGGACAGTCGGGGGCATCAGTATTCAATTGTCAGAGGTG

AAATTCTTGGATTTATTGAAGACTAACTACTGCGAAAGCATTTGCCAAGGATGTTTTCAT

TAATCAGGAACGAAAGTTAGGGGATCGAAGACGATCAGATACCGTCGTAGTCTTAACCAT

AAACTATGCCGATTAGGGATCGGACGGCGTTATTTTTTGACCCGTTCGGCACCTTACGAT

AAATCAAAATGTTTGGGCTCCTGGGGGAGTATGGTCGCAAGGCTGAAACTTAAAGAAATT

GACGGAAGGGCACCACCAGGGGTGGAGCCTGCGGCTTAATTTGACTCAACACGGGGAAAC

TCACCAGGTCCAGACACGATGAGGATTGACAGATTGAGAGCTCTTTCTTGATTTCGTGGG

TGGTGGTGCATGGCCGTTCTTAGTTGGTGGAGTGATTTGTCTGCTTAATTGCGATAACGA

ACGAGACCTTAACCTGCTAAATAGCCCGTATTGCTTTGGCAGTACGCCGGCTTCTTAGAG

GGACTATCGGCTCAAGCCGATGGAAGTTTGAGGCAATAACAGGTCTGTGATGCCCTTAGA

TGTTCTGGGCCGCACGCGCGCTACACTGACAGAGCCAGCGAGTACTCCCTTGGCCGGAAG

GCCCGGGTAATCTTGTTAAACTCTGTCGTGCTGGGGATAGAGCATTGCAATTATTGCTCT

TCAACGAGGAATCCCTAGTAAGCGCAAGTCATCAGCTTGCGTTGATTACGTCCCTGCCCT

TTGTACACACCGCCCGTCGCTACTACCGATTGAATGGCTCAGTGAGGCTTTCGGACTGGC

CCAGAGAGGTCGGCAACGACCACTCAGGGCCGGAAAGTTATCCAAACTCGGTCATTTAGA

GGAAGTAAAAGTCGTAACAAGGTCTCCGTTGGTGAACCAGCGGAGG

**>Podospora anserina S mat+ (FO904938)**

TACCTGGTTGATTCTGCCAGTAGTCATATGCTTGTCTCAAAGATTAAGCCATGCATGTCT

AAGTATAAGCAATTATACAGCGAAACTGCGAATGGCTCATTAAATCAGTTATCGTTTATT

TGATATTACCTTACTACATGGATAACCGTGGTAATTCTAGAGCTAATACATGCAAAAAAT

CCCGACTTCGGAAGGGATGTGTTTATTAGATTAAAAACCAATGCCCTCCGGGGCTCACTG

GTGATTCATAATAACCTCTCGAATCGCACGGCCTTGCGCCGGCGATGGTTCATTCAAATT

TCTGCCCTATCAACTTTCGACGGCTGGGTCTTGGCCAGCCATGGTGACAACGGGTAACGG

GGAGTTAGGGCTCGACTCCGGAGAAGGAGCCTGAGAAACGGCTACTACATCCAAGGAAGG

CAGCAGGCGCGCAAATTACCCAATCCCGACACGGGGAGGTAGTGACAATAAATACTGATA

CAGGGCTCTTTTGGGTCTTGTAATTGGAATGAGTACAATTTAAATCCCTTAACGAGGAAC

AATTGGAGGGCAAGTCTGGTGCCAGCAGCCGCGGTAATTCCAGCTCCAATAGCGTATATT

AAAGTTGTTGAGGTTAAAAAGCTCGTAGTTGAACCTTGGGCCCGGCCCTCCGGTCCCCCT

CACCGGGTGCACTGGCTCGGCCGGGCCTTTCCTTCTGGAGAACCGCATGCCCTTCACTGG

GCGTGCCGGGGAACCAGGACTTTTACTCTGAACAAATTAGATCGCTTAAAGAAGGCCTAT

GCTCGAATAGTCTAGCATGGAATAATGGAATAGGACGTGTGGTTCTATTTTGTTGGTTTC

TAGGACCGCCGTAATGATTAATAGGGACAGTCGGGGGCATCAGTATTCAATTGTCAGAGG

TGAAATTCTTGGATTAATTGAAGACTAACTACTGCGAAAGCATTTGCCAAGGATGTTTTC

ATTAATCAGGAACGAAAGTTAGGGGATCGAAGACGATCAGATACCGTCGTAGTCTTAACC

ATAAACTATGCCGATTAGGGATCGGACGGTGTTATTTTTTGACCCGTTCGGCACCTTGCG

ATAAATCAAAATGTTTGGGCTCCTGGGGGAGTATGGTCGCAAGGCTGAAACTTAAAGAAA

TTGACGGAAGGGCACCACCAGGAGTGGAGCCTGCGGCTTAATTTGACTCAACACGGGGAA

ACTCACCAGGTCCAGACACGATGAGGATTGACAGATTGAGAGCTCTTTCTTGATTTCGTG

GTTGGTGGTGCATGGCCGTTCTTAGTTGGTGGAGTGATTTGTCTGCTTAATTGCGATAAC

GAACGAGACCTTAACCTGCTAAATAGCCCGCATCGCTTTGGCGGTGCGCCGGCTTCTTAG

AGGGACTATCGGCTCAAGCCGATGGAAGTTTGAGGCAATAACAGGTCTGTGATGCCCTTA

GATGTTCTGGGCCGCACGCGCGCTACACTGACAGAGCCAGCGAGTACTCCCTTGGCCGGA

AGGCCCGGGTAATCTTGTTAAACTCTGTCGTGCTGGGGATAGAGCATTGCAATTATTGCT

CTTCAACGAGGAATTCCTAGTAAGCGCAAGTCATCAGCTTGCGCTGATTACGTCCCTGCC

CTTTGTACACACCGCCCGTCGCTACTACCGATTGAATGGCTCAGTGAGGCTTCCGGACTG

GCCCAGGGAGGTCGGCAACGACCACCCAGGGCCGGAAAGCTATCCAAACTCGGTCATTTA

GAGGAAGTAAAAGTCGTAACAAGGTCTCCGTTGGTGAACCAGCGGAGGGATCATTA

**>Neurospora crassa NRRL 13141 (AY046271)**

AGTCATATGCTTGTCTCAAAGATTAAGCCATGCATGTCTAAGTTTAAGCAATTAAACCGC

GAAACTGCGAATGGCTCATTAAATCAGTTATAGTTTATTTGATAGTACCTTACTACATGG

ATAACCGTGGTAATTCTAGAGCTAATACATGCTAAAAACCCCGACTTCGGAAGGGGTGTA

TTTATTAGATTAAAAACCAATGCCCTTCGGGGCTAACTGGTGATTCATAATAACTTCTCG

AATCGCATGGCCTTGCGCTGGCGATGGTTCATTCAAATTTCTGCCCTATCAACTTTCGAC

GGCTGGGTCTTGGCCAGCCATGGTGACAACGGGTAACGGAGGGTTAGGGCTCGACCCCGG

AGAAGGAGCCTGAGAAACGGCTACTACATCCAAGGAAGGCAGCAGGCGCGCAAATTACCC

AATCCCGACACGGGGAGGTAGTGACAATAAATACTGATACAGGGCTCTTTTGGGTCTTGT

AATTGGAATGAGTACAATTTAAATCCCTTAACGAGGAACAATTGGAGGGCAAGTCTGGTG

CCAGCAGCCGCGGTAATTCCAGCTCCAATAGCGTATATTAAAGTTGTTGAGGTTAAAAAG

CTCGTAGTTGAACCTTGGGCTCGGCCCGTCGGTCCGCCTCACCGCGTGCACTGACTGGGT

CGGGCCTTTTTTCCTGGAGAACCGCATGCCCTTCACTGGGTGTGTCGGGGAACCAGGACT

TTTACCGTGAACAAATCAGATCGCTCAAAGAAGGCCTATGCTCGAATGTACTAGCATGGA

ATAATAGAATAGGACGTGTGGTTCTATTTTGTTGGTTTCTAGGACCGCCGTAATGATTAA

TAGGGACAGTCGGGGGCATCAGTATTCAATTGTCAGAGGTGAAATTCTTGGATTTATTGA

AGACTAACTACTGCGAAAGCATTTGCCAAGGATGTTTTCATTAATCAGGAACGAAAGTTA

GGGGATCGAAGACGATCAGATACCGTCGTAGTCTTAACCATAAACTATGCCGATTAGGGA

TCGGACGGTGTTATTTTTTGACCCGTTCGGCACCTTACGATAAATCAAAATGTTTGGGCT

CCTGGGGGAGTATGGTCGCAAGGCTGAAACTTAAAGAAATTGACGGAAGGGCACCACCAG

GGGTGGAGCCTGCGGCTTAATTTGACTCAACACGGGGAAACTCACCAGGTCCAGACACGA

TGAGGATTGACAGATTGAGAGCTCTTTCTTGATTTCGTGGGTGGTGGTGCATGGCCGTTC

TTAGTTGGTGGAGTGATTTGTCTGCTTAATTGCGATAACGAACGAGACCTTAACCTGCTA

AATAGCCCGTATTGCTTTGGCAGTACGCTGGCTTCTTAGAGGGACTATCGGCTCAAGCCG

ATGGAAGTTTGAGGCAATAACAGGTCTGTGATGCCCTTAGATGTTCTGGGCCGCACGCGC

GCTACACTGACACAGCCAGCGAGTACTCCCTTGGCCGGAAGGTCCGGGTAATCTTGTTAA

ACTGTGTCGTGCTGGGGATAGAGCATTGCAATTATTGCTCTTCAACGAGGAATCCCTAGT

AAGCGCAAGTCATCAGCTTGCGTTGATTACGTCCCTGCCCTTTGTACACACCGCCCGTCG

CTACTACCGATTGAATGGCTCAGTGAGGCTTCCGGACTGGCCCAGGGAGGTCGGCAACGA

CCACCCAGGGCCGGAAAGCTATCCAAACTCGGTCATTTAGAGGAAGTAAAAGTCGTAACA

AGGTTT

**>Magnaporthe grisea Ina168 (AB026819)**

TACCTGGTTGATTCTGCCAGTAGTCATATGCTTGTCTTAAAGATTAAGCCATGCATGTCT

AAGTATAAGCAATTATACAGCGAAACTGCGAATGGCTCATTAAATCAGTTATCGTTTATT

TGATAGTACCTTACTACATGGATAACCGTGGTAATTCTAGAGCTAATACATGCTAAAAAC

CCCGACTTCGGAAGGGGTGTATTTATTAGATTAAAAACCAATGCCCTTCGGGGCTCACTG

GTGATTCATGATAACTTCTCGAATCGCACGGCCTTGCGCCGGCGATGGTTCATTCAAATT

TCTGCCCTATCAACTTTCGACGGCTGGGTCTTGGCCAGCCGTGGTGACAACGGGTAACGG

AGGGTTAGGGCTCGACCCCGGAGAAAACGCCTGAGAAACGGCGTTTACATCCAAGGAAGG

CAGCAGGCGCGCAAATTACCCAATGCCGACACGGCGAGGTAGTGACGAGAAATACTGATA

CAGGGCTCTTTTGGGTCTTGTAATTGGAATGAGTACAATTTAAATCTCTTAACGAGGAAC

AATTGGAGGGCAAGTCTGGTGCCAGCAGCCGCGGTAATTCCAGCTCCAATAGCGTATATT

AAAGTTGTTGCAGTTAAAAAGCTCGTAGTTGAACCTTGGGCCTGGCTGGCCGGTCCGCCT

CACCGCGTGCACTGGTCCGGCCGGGCCTTTCCCTCTGGGGAACCGCATGCCCTTCACTGG

GCGTGTCGGGGAACCAGGACTTTTACTTTGAAAAAATTAGAGTGTTCAAAGCAGGCCTAT

GCTCGAATACATTAGCATGGAATAATAGAATAGGACGTGTGGTTCTATTTTGTTGGTTTC

TAGGACCGCCGTAATGATTAATAGGGACAGTCGGGGGCATCAGTATTCAATTGTCAGAGG

TGAAATTCTTGGATTTATTGAAGACTAACTACTGCGAAAGCATTTGCCAAGGATGTTTTC

ATTAATCAGGAACGAAAGTTAGGGGATCGAAGACGATCAGATACCGTCGTAGTCTTAACC

ATAAACTATGCCGACTAGGGATCGGACGGTGTTATTTTTTGACCCGTTCGGCACCTTACA

CGAAAGTACAAGTTTCTGGGTTCTGGGGGGAGTATGGTCGCAAGGCTGAAACTTAAAGAA

ATTGACGGAAGGGCACCACCAGGGGTGGAGCCTGCGGCTTAATTTGACTCAACACGGGGA

AACTCACCAGGTCCAGACATGAAAAGGATTGACAGATTGAGAGCTCTTTCTTGATTTTAT

GGGTGGTGGTGCATGGCCGTTCTTAGTTGGTGGAGTGATTTGTCTGCTTAATTGCGATAA

CGAACGAGACCTTAACCTGCTAAATAGCCCGCGTCGCTTTGGCGGCGCGCCGGCTTCTTA

GAGGGACTATCGGCTCAAGCCGATGGAAGTTTGAGGCAATAACAGGTCTGTGATGCCCTT

AGATGTTCTGGGCCGCACGCGCGCTACACTGACACAGCCAGCGAGTCCTTCCTTGGCCGA

GAGGCCCGGGTAATCTTGTTAAACTGTGTCGTGCTGGGGATAGAGCATTGCAATTATTGC

TCTTCAACGAGGAATCCCTAGTAAGCGCAAGTCATCAGCTTGCGTTGATTACGTCCCTGC

CCTTTGTACACACCGCCCGTCGCTACTACCGATTGAACGGCTCAGTGAGGCCTTCGGACT

GGCCGAGAGAGGTGGGCAACCACCACTCATGTGCCGGAAAGTTGTACGAACTCGGTCGTT

TAGAGGAAGTAAAAGTCGTAACAAGGTCTCCGTTGGTGAACCAGCGGAGGGATCATTA

**>Fusarium graminearum NBRC 9462 (AB250414)**

CATGCATGTCTAAGTATAAGCAATTATACAGCGAAACTGCGAATGGCTCATTATATAAGT

TATCGTTTATTTGATAGTACCTTACTACTTGGATAACCGTGGTAATTCTAGAGCTAATAC

ATGCTAAAAATCCCGACTTCGGAAGGGATGTATTTATTAGATTAAAAACCAATGCCCTCC

GGGGCTCACTGGTGATTCATGATAACTCCTCGAATCGCATGGCCTTGCGCCGGCGATGGT

TCATTCAAATTTCTTCCCTATCAACTTTCGATGTTTGGGTATTGGCCAAACATGGTTGCA

ACGGGTAACGGAGGGTTAGGGCTCGACCCCGGAGAAGGAGCCTGAGAAACGGCTACTACA

TCCAAGGAAGGCAGCAGGCGCGCAAATTACCCAATCCCGACACGGGGAGGTAGTGACAAT

AAATACTGATACAGGGCTCTTTTGGGTCTTGTAATTGGAATGAGTACAATTTAAATCCCT

TAACGAGGAACAATTGGAGGGCAAGTCTGGTGCCAGCAGCCGCGGTAATTCCAGCTCCAA

TAGCGTATATTAAAGTTGTTGTGGTTAAAAAGCTCGTAGTTGAACCTTGGGCCTGGCTGG

CCGGTCCGCCTCACCGCGTGTACTGGTCCGGCCGGGCCTTTCCCTCTGTGGAACCCCATG

CCCTTCACTGGGCGTGGCGGGGAAACAGGACTTTTACTGTGAAAAAATTAGAGTGCTCCA

GGCAGGCCTATGCTCGAATACATTAGCATGGAATAATAGAATAGGACGTGTGGTTCTATT

TTGTTGGTTTCTAGGACCGCCGTAATGATTAATAGGGACAGTCGGGGGCATCAGTATTCA

ATTGTCAGAGGTGAAATTCTTGGATTTATTGAAGACTAACTACTGCGAAAGCATTTGCCA

AGGATGTTTTCATTAATCAGGAACGAAAGTTAGGGGATCGAAGACGATCAGATACCGTCG

TAGTCTTAACCATAAACTATGCCGACTAGGGATCGGACGGTGTTATTTTTTGACCCGTTC

GGCACCTTACGAGAAATCAAAGTGCTTGGGCTCCAGGGGGAGTATGGTCGCAAGGCTGAA

ACTTAAAGAAATTGACGGAAGGGCACCACCAGGGGTGGAGCCTGCGGCTTAATTTGACTC

AACACGGGGAAACTCACCAGGTCCAGACACAATGAGGATTGACAGATTGAGAGCTCTTTC

TTGATTTTGTGGGTGGTGGTGCATGGCCGTTCTTAGTTGGTGGAGTGATTTGTCTGCTTA

ATTGCGATAACGAACGAGACCTTAACCTGCTAAATAGCCCGTATTGCTTTGGCAGTACGC

TGGCTTCTTAGAGGGACTATCGGCTCAAGCCGATGGAAGTTTGAGGCAATAACAGGTCTG

TGATGCCCTTAGATGTTCTGGGCCGCACGCGCGCTACACTGACGGAGCCAGCGAGTACTT

CCTTGTCCGAAAGGTCCGGGTAATCTTGTTAAACTCCGTCGTGCTGGGGATAGAGCATTG

CAATTATTGCTCTTCAACGAGGAATCCCTAGTAAGCGCAAGTCATCAGCTTGCGTTGATT

ACGTCCCTGCCCTTTGTACACACCGCCCGTCGCTACTACCGATTGAATGGCTCAGTGAGG

CGTCCGGACTGGCCCAGAGTGGTGGGCAACTACCGCTCAGGGCCGGAAAGCTCTCCAAAC

TCGGTCATTTAGAGGAAGTAAAAGTCGTAACAAGGTCTCCGTTGGTGAACCAGCGGAGGG

ATCATTA

**>Rhodosporidium toruloides IAM 13469 (D12806)**

AGTCATATGCTTGTCTCAAAGATTAAGCCATGCATGTCTAAGTTTAAGCAATAAACAGTG

AAACTGCGAATGGCTCATTAAATCAGTCATAGTCTATTTGATGGTACCTTACTACATGGA

TAACTGTGGTAATTCTAGAGCTAATACATGCTGAAAAATCCCGACTTCTGGAAGGGATGT

ATTTATTAGATCCAAAACCAACGGCCTTCGGGTCTCCTTGGTGAATCATGATAACTGCTC

GAATCGCATGGCCTTGCGCCGGCGATGCTTCATTCGAATATCTGCCCTATCAACTTTCGA

TGGTAGGATAGAGGCCTACCATGGTGATGACGGGTAACGGGGAATAAGGGTTCGATTCCG

GAGAGAGGGCCTGAGAAACGGCCCTCAGGTCTAAGGACACGCAGCAGGCGCGCAAATTAT

CCCCTGGCAACACTTTGCCGAGATAGTGACAATAAATAACAATGCAGGGCTCTTACGGGT

CTTGCAATTGGAATGAGTACAATTTAAATCCCTTAACGAGGATCAATTGGAGGGCAAGTC

TGGTGCCAGCAGCCGCGGTAATTCCAGCTCCAATAGCGTATATTAAAGTTGTTGCCGTTA

AAAAGCTCGTAGTCGAACTTCGGGCTCTGCAGCCGGTCCGCCTTCTTGGTGTGTACTTGT

TTGGTGGAGCCTTACCTCCTGGTGAACAGCGATGTCCTTCACTGGGTGTCGTTGCAAACC

AGGACGTTTACTTTGAAAAAATTAGAGTGTTCAAAGCAGGCCTTTGCCCGAATACATTAG

CATGGAATAATAGAATAGGACGCGCGTTCCCATTTTGTTGGTTTCTGAGATCGCCGTAAT

GATTAATAGGGATAGTTGGGGGCATTTGTATTCCGTCGTCAGAGGTGAAATTCTTGGATT

GCCGGAAGACAAACTACTGCGAAAGCATTTGCCAAGGATGTTTTCATTGATCAAGAACGA

AGGAAGGGGGATCGAAAACGATTAGATACCGTTGTAGTCTCTTCTGTAAACTATGCCAAT

TGGGGATCGGCACAGGATTTTTAATGACTGTGTCGGCACCCGAAGAGAAATCTTTAAATG

AGGTTCGGGGGGGAGTATGGTCGCAAGGCTGAAACTTAAAGGAATTGACGGAAGGGCACC

ACCAGGTGTGGAGCCTGCGGCTTAATTTGACTCAACACGGGGAAACTCACCAGGTCCAGA

CACAATAAGGATTGACAGATTGATAGCTCTTTCTTGATCTTGTGGTTGGTGGTGCATGGC

CGTTCTTAGTTGGTGGAGTGATTTGTCTGGTTAATTCCGATAACGAACGAGACCTTAACC

TGCTAAATAGACCAGCCGGCTTTGGCTAGCTGCTGTCTTCTTAGAGGGACTATCAGCGTT

TAGCTGATGGAAGTTTGAGGCAATAACAGGTCTGTGATGCCCTTAGATGTTCTGGGCCGC

ACGCGCGCTACACTGACAGAGCCAGCGAGTCTACCACCTTTGCCGGAAGGCATGGGTAAT

CTTGTGAAACTCTGTCGTGATGGGGATAGAGCATTGCAATTATTGCTCTTCAACGAGGAA

TACCTAGTAAGCGTGATTCATCAGATCGCGTTGATTACGTCCCTGCCCTTTGTACACACC

GCCCGTCGCTACTACCGATTGAATGGCTTAGTGAGGCCTCCGGATTGGCTATTGGGAGCT

CGCGAGAGCACCTGACTGCCGAGAAGTTGTACGAACTTGGTCATTTAGAGGAAGTAAAAG

TCGTAACAAGGTTTCCGTA

**>Sporisorium reilianum AFTOL-ID 490 (DQ832229)**

AATTCGTAGTCATATGCTTGTCTCAAAGATTAAGCCATGCATGTCTAAGTATACGCAAAT

TATACTGTGAAACTGCGAATGGCTCATTAAATCAGTTATAGTTTATTTGATGTTTCTTGC

TACATGGATAACTGTGGTAATTCTAGAGCTAATACATGCGTAAAAAGCCCCGACTTCTGG

AAGGGGTGTATTTATTAGATAAAAACCATCCTCCTCGGAGTTTGGTGATTCATAATAACT

TCTCGAATCGCACGGCCTTGTGCTGGCGATGCTTCATTCAAATATCTGCCCTATCAACTG

TCGATGGTAGGATAGAGGCCTACCATGGTTGCAACGGGTAACGGGGAATAAGGGTTCGAT

TCCGGAGAGGGAGCCTGAGAAACGGCTACCACATCCAAGGAAGGCAGCAGGCGCGCAAAT

TACCCAATCCCGACACGGGGAGGTAGTGACAATAAATAACAATGCAGGGCCCTTTTGGGT

CTTGTAATTGGAATGAGTACAATTTAAATCCCTTAACGAGGAACGATTGGAGGGCAAGTC

TGGTGCCAGCAGCCGCGGTAATTCCAGCTCCAATAGCGTATATTAAAGTTGTTGCAGTTA

AAAAGCTCGTAGTTGAAGTTTGGTCTCGGACGCTGGGTCTGCTTAATTGCATGTACTTGA

CGGTCCGAGACTTCCTTCTTGGTGAACGGCCGCCTTCGGGTGGTCCGGAACCAGGACTAT

TACTTTGAAAAAATTAGAGTGTTCAAAGCAGGCCATAGGCCCGAATATATTAGCATGGAA

TAACAGAATAGGACGTGCGGTTCTATTTTGTTGGTTTCTAGAACTGCCGTAATGATTAAA

AGGGACAGCCGGGGGCATTAGTATTTGCACGCTAGAGGTGAAATTCTTGGATTGTGCAAA

GACTTCCTACTGCGAAAGCATTTGCCAAGAATGTTTTCATTAATCAAGAACGAAGGTTAG

GGTATCGAAAACGATTAGATACCGTTGTAGTCTTAACAGTAAACTATGCCGACTCCGAAT

CGGTCGATGCTCATTTCACTGGCTCGATCGGCGCGGTACGAGAAATCAAAGTTTTTGGGT

TCTGGGGGGAGTATGGTCGCAAGGCTGAAACTTAAAGAAATTGACGGAAGGGCACCACCA

GGAGTGGAGCCTGCGGCTTAATTTGACTCAACACGGGAAAACTCACCGGGTCCGGACATA

GTAAGGATTGACAGATTGATGGCGCTTTCATGATTCTATGGGTGGTGGTGCATGGCCGTT

CTTAGTTGGTGGAGTGATTTGTCTGGTTAATTCCGATAACGAACGAGACCTTGACCTGCT

AAATAGACGGGTTGACATTTTGTTGGCCCCTTATGTCTTCTTAGAGGGACAATCGACCGT

CTAGGTGATGGAGGCAAAAGGCAATAACAGGTCTGTGATGCCCTTAGATGTTCCGGGCTG

CACGCGCGCTACACTGACAGAGACAACGAGTGGGGCCCCTTGTCCGAAATGACTGGGTAA

ACTTGTGAAACTTTGTCGTGCTGGGGATGGAGCTTTGTAATTTTTGCTCTTCAACGAGGA

ATTCCTAGTAAGCGCAAGTCATCAGCTTGCGTTGACTACGTCCCTGCCCTTTGTACACAC

CGCCCGTCGCTACTACCGATTGAATGGCTTAGTGAGGACTTGGGAGAGTACATCGGGGAG

CCAGCAATGGCACCCTGACGGCTCAAACTCTTACAAACTTGGTCATTTAGAGGAAGTAAA

AGTCGTAACAAGGTATCCGTAG

**>Schizophyllum commune (X54865)**

TACCTGGTTGATCCTGCCAGTAGTCATATGCTTGTCTCAAAGATTAAGCCATGCATGTCT

AAGTATAAACAAGTTTGTACTGTGAAACTGCGAATGGCTCATTAAATCAGTTATAATTTA

TTTGATGATACCTTGCTACATGGATAACTGTGGTAATTCTAGAGCTAATACATGCAATCA

AGCCCCGACTTCTGGAAGGGGTGTATTTATTAGATAAAAAACCAACGCGGCTCGCCGCTC

ACTTGGTGATTCATAATAACTTCTCGAATCGCATGGCCTTGCGCCGGCGATGCTTCATTC

AAATATCTGCCCTATCAACTTTCGATGGTAGGATAGAGGCCTACCATGGTTTCAACGGGT

AACGGGGAATAAGGGTTCGATTCCGGAGAGGGAGCCTGAGAAACGGCTACCACATCCAAG

GAAGGCAGCAGGCGCGCAAATTACCCAATCCCGACACGGGGAGGTAGTGACAATAAATAA

CAATATAGGGCTCTTTCGGGTCCTATAATTGGAATGAGTACAATTTAAATCCCTTAACGA

GGATCAATTGGAGGGCAAGTCTGGTGCCAGCAGCCGCGGTAATTCCAGCTCCAATAGCGT

ATATTAAAGTTGTTGCAGTTAAAAAGCTCGTAGTTGAACTTCAGGCCTGGCCGGGCGGTC

TGCCTAACGGTATGTACTGTCTGGCCGGGTCTTACCTCTTGGTGAACCGGCGTGCTCTTT

ACTGGGCGCGTCGGCGAACCAGGACTTTTACCTTGAGAAAATTAGAGTGTTCAAAGCAGG

CTTACGCCCGAATACATTAGCATGGAATAATAAAATAGGACGTGCGGTCCTATTTTGTTG

GTTTCTAGGATCGCCGTAATGATTAATAGGGATAGTTGGGGGCATTGGTATTGAGTCGCT

AGAGGTGAAATTCTTGGATTGACTCAAGACCGACTACTGCGAAAGCATTTGCCAAGGATG

TTTTCATTAATCAAGAACGAAGGTTAGGGGATCGAAAACGATCAGATACCGTTGTAGTCT

TAACAGTAAACTATGCCGACTAGGGATCGGACGACCTCAATTATTATGTGTCGTTCGGCA

CCTTACGAGAAATCAAAGTCTTTGGGTTCTGGGGGGAGTATGGTCGCAAGGCTGAAACTT

AAAGGAATTGACGGAAGGGCACCACCAGGTGTGGAGCCTGCGGCTTAATTTGACTCAACA

CGGGGAAACTCACCAGGTCCAGACATAACTAGGATTGACAGATTGATAGCTCTTTCATGA

TTTTATGGGTGGTGGTGCATGGCCGTTCTTAGTTGGTGGAGTGATTTGTCTGGTTAATTC

CGATAACGAACGAGACCTTAACCTGCTAAATAGCCAGGCCGGCTTTTGCTGGTCTTATGG

CTTCTTAGAGGGACTGTAGGCGTCTAGCTTACGGAAGTTTGAGGCAATAACAGGTCTGTG

ATGCCCTTAGATGTTCTGGGCCGCACGCGCGCTACACTGACAGAGGCAGCGAGTTCTTTT

CCTTGGCCGGAAGGTCCGGGTAATCTTGTGAAACTCTGTCGTGCTGGGGATAGAGCATTG

CAATTATTGCTCTTCAACGAGGAATACCTAGTAAGCGTGAGTCATCAGCTCGCGTTGATT

ACGTCCCTGCCCTTTGTACACACCGCCCGTCGCTACTACCGATTGAATGGCTTAGTGAGG

TCTTCGGATCGGCTTTGGGGAGCCGGCAACGGCACCTCATTGCTGAGAAGTTGATCAAAC

TTGGTCATTTAGAGGAAGTAAAAGTCGTAACAAGGTTTCCGTAGGTGAACCTGCGGAAGG

ATCATTA

**>Piriformospora indica DSM 11827 (AY293147)**

CCAAGCTTGAATTCGTAGTCATATGCTTGTCTCAAAGATTAAGCCATGCATGTCTAAGTA

TAAACAACTTTGTACTGTGAAACTGCGAATGGCTCATTAAATCAGTTATAGTTTATTTGA

TGGTACCTTACTACATGGATAACTGTGGTAATTCTAGAGCTAATACATGCGTCAAAGCCC

CATCCGGGGTGTATTTATTAGATAAAAAACCAACGCGGTTTACCGCTCCCTTGGTGATTC

ATAATAACTTCTCGAATCGCATGGCCTTGTGCCGGCGATGCTTCATTCAAATATCTGCCC

TATCAACTTTCGATGGTAGGATAGAGGCCTACCATGGTTTCAACGGGTAACGGGGAATAA

GGGTTCGATTCCGGAGAGGGAGCCTGAGAAACGGCTACCACATCCAAGGAAGGCAGCAGG

CGCGCAAATTACCCAATCCCGACACGGGGAGGTAGTGACAATAAATAACAATATAGGGCT

CTATTGGGTCTTATAATTGGAATGAGTACAATTTAAACCCCTTAACGAGGAACAATTGGA

GGGCAAGTCTGGTGCCAGCAGCCGCGGTAATTCCAGCTCCAATAGCGTATATTAAAGTTG

TTGCAGTTAAAAAGCTCGTAGTTGAACTTCGAACCTGGTTAGGTGGTCTGCCTTACGGTA

CGTACTGCTCGGCCGGGTTCTACCTCTTGGTGAGCCGGCATGTCCTTCACTGGATGTGTC

GGGGAACCAGGACTTTTACCTTGAGAAAATTAGAGTGTTCAAAGCAGGCCTTTGCCCGAA

TACATTAGCATGGCATAATAAAATAGGACGTGCGGTTCTATTTTGTTGGTTTCTAGAGTC

GCCGTAATGATTAATAGGGATAGTTGGGGGCATTTGTATTCCGTTGCTAGAGGTGAAATT

CTTGGATTTACGGAAGACAAACTTCTGCGAAAGCATTTGCCAAGGATGTTTTCATTAATC

AAGAACGAAGGTTAGGGGATCGAAAACGATCAGATACCGTTGTAGTCTTAACAGTAAACT

ATGCCGACTAGGGATCGGGCGATCTCAATCTTATGTGTCGCTCGGCACCTTACGAGAAAT

CAAAGTCTTTGGGTTCTGGGGGGAGTATGGTCGCAAGGCTGAAACTTAAAGGAATTGACG

GAAGGGCACCACCAGGCGTGGAGCCTGCGGCTTAATTTGACTCAACACGGGGAAACTCAC

CAGGTCCAGACATAACTAGGATTGACAGATTGATAGCTCTTTCTTGATTTTATGGGTGGT

GGTGCATGGCCGTTCTTAGTTGGTGGAGTGATTTGTCTGGTTAATTCCGATAACGAACGA

GACCTTAACCTGCTAAATAGCCAGACCGTCTTTTGATGGTTGCAGGCTTCTTAGAGGGAC

TGTCAGGATCTACCTGACGGAAGTTTGAGGCAATAACAGGTCTGTGATGCCCTTGACCGA

TGATACGTTCACTTTTCGTATTAACATCTAGGGGTAAAAAGTAGGCTTAAAAGGTCTGCT

AGTGATGATGTCTCGGCATCATTGCAACACGTTCAAATTGCGGGAAATTCCTAAAGCTCT

GATTACCGTCTGAGAGTGTGAAAGCCTCTCTGACACCGGGTTAATTGCCCTGGGTATGGT

AAAAAGATCAGAGATGTCACAATGGACAATCCGCAGCGAAGTCCGCTGATCTATACGATC

GTGGAGCGCGTTCAGAGACTAGATGTTCGTGGGCTGCCTCTCAATGGGGTGGCTTAAGGT

ATAGTCCTTCCCGACTCGAAAGGGTTTGGGGTTTAAAGTAGATGTTATGGGCCGCACGCG

CGCTACACTGACAAAGCCAGCGAGTTTATCACCTTGGCCGGAAGGTCCGGGTAATCTTGT

GAAACTTTGTCGTGCTGGGGATAGAGCATTGCAATTATTGCTCTTCAACGAGGAATGCTT

AGTAAGCGCAAGTCATCAGCTTGCGTTGATTACGTCCCTGCCCTTTGTACACACCGCCCG

TCGCTACTACCGATTGAACGGCTTAGTGAGGTCTTCGGATTGGCTTTGGGGATCCGGCAA

CGGAACCCCG

**>Phanerochaete chrysosporium FPL 5175 (AF026593)**

AGCTGAATTCGTAGTCATATGCNTGTCTCAAAGANTAAGCCATGCATGTCTAAGTATAAA

CAAGTTTGTACTGTGAAACTGCGAATGGNTCATTAAATCAGTTATAGTTTATTTGATGGT

GCTTTGCTACATGGATAACTGTGGTAATTCTAGAGCTAATACATGCAATCAAGCCCCGAC

TTCTGGAAGGGGTGTATTTATTAGATAAAAAACCAACGCGGTTCGCCGCTCCATTGGTGA

TTCATAATAACTTCTCGAATCGCATGGCCTTGTGCCGGCGATGCTTCATTCAAATATCTG

CCCTATCAACTTTCGATGGTAGGATAGAGGCCTACCATGGTTTCAACGGGTAACGGGGAA

TAAGGGTTCGATTCCGGAGAGGGAGCCTGAGAAACGGCTACCACATCCANGGAAGGCAGC

AGGCGCGCAAATTACCCAATCCCGACACGGGGAGGTAGTGACAATAAATAACAATATAGG

GCTCTTTCGGGTCTTATAATTGGAATGAGTACAATTTAAATCTCTTAACGAGGAACAATT

GGAGGGCAAGTCTGGTGCCAGCAGCCGCGGTAATTCCAGCTCCAATAGCGTATATTAAAG

TTGTTGCAGTTAAAAAGCTCGTAGTTGAACTTCAGGCCTGGCTGGGCGGTCTGCCTCACG

GTATGTACTGTCTGGCTGGGTCTTACCTCTTGGTGAGCCGGCATGCCCTTCACTGGGTGT

GTCGGGGAACCAGGACTTTTACCTTGAGAAAATTAGAGTGTTCAAAGCAGGCTTATGCCC

GAATACATTAGCATGGAATAATAAAATAGGACGTGCGGTTCTATTTTGTTGGTTTCTAGA

GTCGCCGTAATGATTAATAGGGATAGTTGGGGGCATTAGTATTCCGTTGCTAGAGGTGAA

ATTCTTGGATTTACGGAAGACTAACTACTGCGAAAGCATTTGCCAAGGATGTTTTCATTA

ATCAAGAACGAAGGTTAGGGGATCGAAAACGATCAGATACCGTTGTAGTCTTAACAGTAA

ACTATGCCGACTAGGGATCGGGCGAACTCAATTTGATGTGTCGCTCGGCACCTTACGAGA

AATCAAAGTACTTTGGGTTCTGGGGGGAGTATGGTCGCAAGGCTGAAACTTAAAGGAATT

GACGGAAGGGCACCACCAGGTGTGGAGCCTGCGGCTTAATTTGACTCAACACGGGGAAAC

TCACCAGGTCCAGACATGACTAGGATTGACAGATTGATAGCTCTTTCATGATTTTATGGG

TGGTGGTGCATGGCCGTTCTTAGTTGGTGGAGTGATTTGTCTGGTTAATTCCGATAACGA

ACGAGACCTTAACCTGCTAAATAGCCAGGCCGGCTTTTGCTGGTCGCCGGCTTCTTAGAG

GGACTGTCTGCGTCTAGCAGACGGAAGTTTGAGGCAATAACAGGTCTGTGATGCCCTTAG

ATGTTCTGGGCCGCACGCGCGCTACACTGACAGAGCCAGCGAGTTTTTTTCCTTGGCCGG

AAGGTCTGGGTAATCTTGTGAAACTCTGTCGTGCTGGGGATAGAGCATTGCAATTATTGC

TCTTCAACGAGGAATACCTAGTAAGCGTGAGTCATCAGCTCGCGTTGATTACGTCCCTGC

CCTTTGTACACACCGCCCGTCGCTACTACCGATTGAATGGCTTAGTGAGGCCTTGGGATT

GGCTTCGGGGAGCCGGCAACGGCACCCTGTTGCTGAGAACTTGGTCAAACTTGGTCATTT

AGAGGAAGTAAAAGTCGTAACAAGGTTTCCGTAGGTGAACCTGCGGAAGGATCATTAACN

ANTAAC

**>Postia placenta Mad-698-R (ABWF01010551)**

AACGGTATGTACTGTCTGGCTGGGTCTTACCTCTTGGTGATCCGGCATGTCCTTTACTGG

GTGTGTCGGGGAACCAGGACCTTTACCTTGAGAAAATTAGAGTGTTCAAAGCAGGCCTGT

GCCCGAATACATTAGCATGGAATAATAAAATAGGACGTGCGGTTCTATTTTGTTGGTTTC

TAGAGTCGCCGTAATGATTAATAGGGATAGTTGGGGGCATTAGTATTCAGTTGCTAGAGG

TGAAATTCTTGGATTTACTGAAGACTAACTACTGCGAAAGCATTTGCCAAGGATGTTTTC

ATTAATCAAGAACGAAGGTTAGGGGATCGAAAACGATCAGATACCGTTGTAGTCTTAACA

GTAAACTATGCCGACTAGGGATCGGGCGATCTCAATTTTATGTGTCGCTCGGCACCTTAC

GAGAAATCAAAGTCTTTGGGTTCTGGGGGGAGTATGGTCGCAAGGCTGAAACTTAAAGGA

ATTGACGGAAGGGCACCACCAGGTGTGGAGCCTGCGGCTTAATTTGACTCAACACGGGGA

AACTCACCAGGTCCAGACATGACTAGGATTGACAGATTGATAGCTCTTTCATGATTTTAT

GGGTGGTGGTGCATGGCCGTTCTTAGTTGGTGGAGTGATTTGTCTGGTTAATTCCGATAA

CGAACGAGACCTTAACCTGCTTAATAGTCAGGCCGGCTTTTGCTGGTCGCCGACTTCTTA

GAGGGACTGTCTGCGTCTAGCAGACGGAAGTTTGAGGCAATAACAGGTCTGTGATGCCCT

TAGATGTTCTGGGCCGCACGCGCGCTACACTGACAGAGCCAGCGAGTATTTTTCCTTGGC

CGGAAGGTCTGGGTAATCTTGTGAAACTCTGTCGTGCTGGGGATAGAGCATTGCAATTAT

TGCTCTTCAACGAGGAATACCTAGTAAGCGTGAGTCATCAGCTCGCGTTGATTACGTCCC

TGCCCTTTGTACACACCGCCCGTCGCTACTACCGATTGAATGGCTTAGTGAGGTCTTGGG

ATT

**>Rhizopus oryzae KCTC46312 (GDUK010232540**

TCGAGGCTGTGAGTCTTGGATAACCTATGGTAGAAAGGAAATATCTTTTCTACTGTTAAA

GTTCCCGGATTAAATCTTGTCGTACACTTCCTTATGGGAGCAGATGGGCGAGTCGCTGGC

TCCTGCGGAAGCTCTTTGAGTTACCGTAGTGAGAAAAGATGGGGATTGTATATTATTACC

TATCCAGGTATGATTACAAGCCAACTCCTGGGCACCTTTATTGGAGTCCATCGACTGATC

TGCTGGGAAAAATTTATTTTTCTTTGCGTTGATCGGACGAAAACTGTAGGATTGCTAAAG

GGAAATTAAAGTAGATTGTGCAAACGTTCAGCAGATATGCAGAATGTAGTATGATCTGCT

TTCTCTTTCAAAGGGTTTATCCCCTTTGGGTAGTCGACTGGTACGCCATGGAAAAAAAGT

GGGCTCTTCTTTGAAGAGTCTCGTCTAAGCTTTCGAGTTTAGGCTAACTTTTTAACCTGA

TAGTTACCTGGTTGATCCTGCCAGTAGTCATATGCTTGTCTCAAAGATTAAGCCATGCAT

GTCTAAGTATAAATAACTTTATATTGTGAAACTGCGAATGGCTCATTAAATCAGTTATGA

TCTACGTGACAAATTCTTTACTACTTGGATAACCGTGGTAATTCTAGAGCTAATACATGC

AAAAAAGCCCTGACTTACGAAGGGGTGCACTTATTAGATAAAACCAACGCGGGGTAAAAC

CTGTTTCTTGGTGAATCATAATAATTAAGCGGATCGCATGGCCTTGTGCCGGCGACGGTC

CACTCGATTTTCTGCCCTATCATGGTTGAGATTGTAAGATAGAGGCTTACAATGCCTACA

ACGGGTAACGGGGAATTAGGGTTCGATTCCGGAGAGGGAGCCTGAGAAACGGCTACCACA

TCCAAGGAAGGCAGCAGGCGCGCAAATTACCCAATCCCGACACGGGGAGGTAGTGACAAT

ACATAACAATGCAGGGCCTTTAAGGTCTTGCAATTGGAATGAGTACAATTTAAATCCCTT

AACGAGGATCAATTGGAGGGCAAGTCTGGTGCCAGCAGCCGCGGTAATTCCAGCTCCAAT

AGCGTATATTAAAGTTGTTGCAGTTAAAACGTCCGTAGTCAAACTTTAGTCTTACCGGCG

TAGTGGCCTGGTCTTCATTGACCAAGCTCATTGCTGCCGGAGACTCCATGTCCATTGACT

CCTAGTCCTCGTGGCTAGGGTTTTCTGGACAATTACCATGAGCAAATCAGAGTGTTTAAA

GCAGGCTTTTAAGCTTGAATGTGTTAGCATGGAATAATGAAATATGACTTTAGTCCTATT

TTCGTTGGTTTAGGTACTTCAGTAATGATGAATAGAAACGGTTAGGGGCATTTGTATTTG

GTCGCTAGAGGTGAAATTCTTGGATTGACCGAAGACAAACTACTGCGAAAGCATTTGACC

CGGGACGTTTTCATTGATCAAGGTCTAAAGTTAAGGGATCGAAGACGATTAGATACCGTC

GTAGTCTTAACCACAAACTATGCCGACTAGAGATTGGGCGTGTTTATTATGACTCGCTCA

GCATCTTAGCGAAAGTAAAGTTTTTGGGTTCTGGGGGGAGTATGGGACGCAAGGCTGAAA

CTTAAAGGAATTGACGGAAGGGCACCACCAGGAGTGGAGCCTGCGGCTTAATTTGACCCA

ACACGGGGAAACTCACCAGGTCCAGACATAGTAAGGATTGACAGATTGAAAGCTCTTTCT

AGATTCTATGGGTGGTGGTGCATGGCCGTTCTTAGTTCGTGGAGTGATTTGTCTGGTTAA

TTCCGATAACGAACGAGACCTTATTCTGCTAATTAGACAGGCTAACTCTTTCGGGTTGGT

TTATATTTAATATTTAACTGGCTTCTTAGAGAGACTATCGGCTTCAAGCCGAAGGAAGTT

TTAGGCAATAACAGGTCTGTGATGCCCTTAGATGTTCTGGGCCGCACGCGCGCTACACTG

ATGAAGTCAGCGAGTTTATAACCTTGGCCGGAAGGTCTGGGTAAACTTTTGAAACTTCAT

CGTGCTGGGGATAGAGCATTGTAATTATTGCTCTTCAACGAGGAATTCCTAGTAAGCGCA

AGTCATCAGCTTGCGTTGATTACGTCCCTGCCCTTTGTACACACCGCCCGTCGCTACTAC

CGATTGAATGGTTATAGTGAGCATATGGGATCAGTAGGATTTGACTGGCAACAGTCATTT

CCTGCAGAGAACTATGGCAAACTAGGCTATTTAGAGGAAGTAAAAGTCGTAACAAGGTTT

CCGTAGGTGAACCTGCGGAAGGATCATTAATTATGTTAAAGCGCCTTACCTTAGGGTTTC

CTCTGGGGTAAGTGATTGCTTCTACACTGTGAAAATTTGGCTGAGAGACTCAGACTGGTC

ATGGGTAGACCTATCTGGGGTTTGATCGATGCCACTCCTGGTTTCAGGAGCACCCTTCAT

AATAAACCTAGAAATTCAGTATTATAAAGTTTAATAAAAAACAACTTTTAACAATGGATC

TCTTGGTTCTCGCATCGATG

**>Phytophthora infestans TD-2 (AY742761)**

GTAGTCATATGCTTGTCTCAAAGATTAAGCCTTGCATGTCTAAGTATAAACAATTTTGTA

CTGTGAAACTGCGAATGGCTCATTATATCAGTTATAGTCTACTCGATAGTACCTTACTAC

TTGGATACCCGTAGTAATTCTAGAGCTAATACATGCATCAATACCCAACTGCTTGTCGGG

CGGGTAGCATTTATTAGATTGAAACCAATGCAGTCTTCGGGCTGGTATTGTGTTGAGTCA

TAATAACTGTGCGGATCGCGCTTTTGCGCGATAAATCGATTGAGTTTCTGCCCTATCAGC

TTTGGATGGTAGGATATGGGCCTACCATGGCATTAACGGGTAACGGGGAATTAGGGTTTG

ATTCCGGAGAGGGAGCCTTAGAATCGGCTACCACATCCAAGGAAGGCAGCAGGCGCGTAA

ATTACCCAATCCTGACACAGGGAGGTAGTGACAATAAATAACAATGCTCTGGCTCTTCGA

GTCGGGCAATTGGAATGAGAACAATTTAAATCCCTTAACGAGGATCAATTGGAGGGCAAG

TCTGGTGCCAGCAGCCGCGGTAATTCCAGCTCCAATAGCGTATATTAAAGTTGTTGCAGT

TAAAAAGCTCGTAGTTGGATTTCTGTTTTGGATGTCCGGTCCGCTCCCTCTGGGAGTGTG

TACTTATGGATATTCGAGGCATTTTTTGTGAGGCTGCCTTTCTGCCATTAAGTTGGTGGG

TTGGTGGGCTTGCATCGTTTACTGTGAAAAAATTAGAGTGTTTAAAGCAGGCGTTTGCTC

ATTTGAATACATTAGCATGGAATAATAAGATACGGCCTTGGTGGTCTATTTTGTTGGTTT

GCACACCAGGGTAATGATTAATAGGGACAGTTGGGGGTATTCATATTTCAGCGTCAGAGG

TGAAATTCTTGGATCGCTGAAAGATGAGCTTAGGCGAAAGCATTTACCAAGGACGTTTTC

ATTAATCAAGAACGAAAGTTAGGGGATCGAAGATGATTAGATACCATCGTAATCTTAACC

ATAAACTATGCCGACTCGGGATTGGCAGTCGTTTACTTTAAATGACCTTGTCAGCACCGT

ATGAGAAATCAAAGTCTTTGGGTTCCGGGGGGAGTATGGTCGCAAGGCTGAAACTTAAAG

GAATTGACGGAAGGGCACCACCAGGAGTGGAGCCTGCGGCTTAATTTGACTCAACACGGG

AAAACTTACCAGGTCCAGACATAGTAAGGATTGACAGACTGAGAGCTCTTTCTTGATTCT

ATGGGTGGTGGTGCATGGCCGTTCTTAGTTGGTGGAGTGATTTGTCTGGTTAATTCCGTT

AACGAACGAGACCTCCGCGTGCTAAATAGTTCCGCTTACCATTTTTGGTAGGTTTGTGGA

CTTCTTAGAGGGACTTTTGGGTAATCAAACCAAAGGAAGTTGGAGGCAATAACAGGTCTG

TGATGCCCTTAGATGTTCTGGGCCGCACGCGCGCTACACTGATGCGTTCAACGAGTATAC

AACCTTGATCGATAGGTCTGGGTAATCTTGTGAATGCGCATCGTGCTAGGGATAGACTGT

TGCAATTTTCAGTCTTGAACGAGGAATTCCTAGTAAACGCAAGTCATCTGCTTGCATTGA

TTACGTCCCTGCCCTTTGTACACACCGCCCGTCGCACCTACCGATTGAATGACTCGGTGA

AAAATTGGGACCGTGAGTCTGTTTGCTTTATTGCGAGTGGATTGATGGGAACTTTTTTAA

ACCTCGCCATTTAGAGGAAGGTGAAGTCGTAACAAGGTTTCCGTAGGTGAACCTGCGGAA

GGATCA

**References**

1. Esteve-Zarzoso B, Belloch C, Uruburu F, Querol A. Identification of yeasts by RFLP analysis of the 5.8S rRNA gene and the two ribosomal internal transcribed spacers. Int. J. Syst. Bacteriol. 1999;49:329–37.

2. Tamura K, Nei M. Estimation of the number of nucleotide substitutions in the control region of mitochondrial DNA in humans and chimpanzees. Mol. Biol. Evol. 1993;10:512–26.

3. Kumar S, Stecher G, Tamura K. MEGA7: Molecular Evolutionary Genetics Analysis version 7.0 for bigger datasets. Mol. Biol. Evol. 2016;msw054.
